# Exogenous putrescine modulates variety-specific cadmium tolerance in wheat seedlings: synergistic roles of antioxidant defense and physiological homeostasis

**DOI:** 10.3389/fpls.2025.1600603

**Published:** 2025-09-08

**Authors:** Dandan Zhong, Haili Yan, Xinxia Chen, Ziyu Zhong, Xuerui Li, Xiangzhen Jia, Siyu Chang, Jie Shen, Dongxu Zhang

**Affiliations:** ^1^ College of Agriculture, Shanxi Agricultural University, Jinzhong, China; ^2^ Millet Research Institute, Shanxi Agricultural University, Changzhi, China; ^3^ Department of Life Sciences, Changzhi University, Changzhi, China; ^4^ Key Laboratory of Sustainable Dryland Agriculture (Co-construction by Ministry and Province), Ministry of Agriculture and Rural Affairs, Jinzhong, China

**Keywords:** wheat seedlings, Cd stress, exogenous putrescine, oxidative stress, osmotic regulation

## Abstract

**Introduction:**

Cadmium (Cd) contamination in farmland is a significant environmental issues affecting crop yields. Putrescine (Put), a polyamine compound, functions as a signaling molecule that actively mediates plant responses to environmental adversities. Wheat exhibits a distinctive propensity for translocating the heavy metal Cd into its seeds compared to other crops, which poses a critical environmental adaptation challenge that needs to be addressed in agricultural systems.

**Methods:**

This study employed Changmai 4013 (Cd-tolerant) and Chang 6475 (Cd-sensitive) varieties as test materials to investigate the regulatory effect of exogenous Put treatment on the Cd tolerance of both varieties under Cd stress. Wheat seeds were soaked in 0.05, 0.1, 0.2, 0.4, 0.8, and 1.6 mM Put solution, and then cultured in a 80 mg·L^−1^ Cd solution.

**Results:**

The results indicated that Cd stress significantly inhibited wheat germination and seedling vigor. However, exogenous Put treatment effectively alleviated the stress-induced damage. It reduced malondialdehyde (MDA) and hydrogen peroxide (H_2_O_2_) content, decreased relative electrical conductivity, increased catalase (37.4%), glutathione (39.47%), and relative water content (30.67%), promoted the synthesis of osmotic regulators, reduced Cd accumulation in roots and shoots, and promoted growth. Exogenous Put also significantly increased the endogenous levels of spermidine (Spd), spermine (Spm), and Put in both cultivars. Significant cultivar differences were observed in the response, as polyamine levels in Changmai 4013 peaked at the 0.1 mM Put treatment, while Chang 6475 showed the most significant increase in endogenous Put content at the 0.2 mM Put treatment. A comprehensive evaluation using the Membership Function (MV) method indicated that the 0.1 mM Put treatment provided the best overall alleviation effect for both cultivars.

**Discussion:**

Multivariate analysis revealed distinct mitigation mechanisms between the two cultivars. Changmai 4013 primarily relied on maintaining physiological homeostasis, whereas Chang 6475 depended on enhancing the antioxidant system. Furthermore, the latter exhibited a stronger demand for and utilization capacity of exogenous Put. These findings provide an important theoretical basis for wheat cultivar selection in Cd-contaminated areas and the precision field application of Put.

## 1 Introduction

Wheat (*Triticum aestivum* L.) is a key cereal crop globally, serving as a primary food staple for many nations and regions (Beres et al., 2020). As agricultural technology advances, mechanized sowing has become a popular method for wheat planting (Muhammad et al., 2024). However, this sowing method demands stricter seed quality requirements. Therefore, improving seed resistance and seedling viability is essential to ensure sustained high yields (Zhang et al., 2024). The emergence and seedling stages are critical in wheat development and are highly susceptible to abiotic stress. Currently, many studies have found that it is possible to modulate the negative effects caused by these environmental factors through the use of exogenous substances to improve final yields and quality (Liu et al., 2023).

Cadmium (Cd) is a heavy metal pollutant with high toxicity in soil (Xia et al., 2024), which usually exists in soil in ionic form, and due to its high bioavailability and strong mobility, it can be transported to various parts of the plant through the roots (Riaz et al., 2021), making plants highly sensitive to excessive Cd accumulation. This not only damages plant cell membranes but also induces a surge in reactive oxygen species (ROS) within, which significantly disrupts the ultrastructural organization of the organelles (Zhang et al., 2023). Meanwhile, excess Cd accumulates in crop fruits and is transmitted to people through food chains via bioconcentration (Liu et al., 2020). Wheat is more likely to accumulate Cd and transfer it to the seed site than other cereal crops (Jafarnejadi et al., 2011), and this issue has become a critical challenge for both environmental sustainability and food security worldwide.

Polyamines (PAs) are considered as growth regulators or secondary messengers (Doneva et al., 2021). Common PAs in plants are organic compounds such as putrescine (Put), spermine (Spm), and spermidine (Spd), which play key roles in plant growth and development, and have been used in studies of a variety of environmental stresses (Pramanick et al., 2022; Hai et al., 2022; Yang et al., 2022). It was found that 0.1 mM Spd promotes the synthesis of osmoregulatory substances in ramie, and these effects have been widely studied for their role in plant responses to environmental stresses (Gong et al., 2016). At the same time, 2 mM Spm and Spd could also help to remove excessive ROS from wheat in time to ameliorate the damage suffered by wheat, and both of them alleviated Cd stress to a similar extent (Rady and Hemida, 2015). Other research indicated that 0.5 mM Spd can reduce electrical conductivity and MDA toxicity in seedlings, alleviating cell membrane damage and protecting rice from Cd and Pb toxicity (Gu et al., 2022). It is noteworthy that under aluminum stress, the 0.5 mM Spd treatment also demonstrated beneficial regulatory effects. Not only did it activate the key PAs biosynthesis enzyme arginine decarboxylase (ADC) and increase PAs content in chloroplasts, but it also enhanced the activity of antioxidant enzymes in rice chloroplasts, thereby significantly reducing H_2_O_2_ accumulation (Jiang et al., 2021). Furthermore, 0.5 mM Put has also been demonstrated to promote the morphological development of mustard seedlings and alleviate Cd toxicity through mechanisms including osmotic regulation, antioxidant enzyme activation, and improved nutrient absorption (Chowardhara et al., 2022). These findings demonstrate that optimal PAs concentrations strengthen plant stress resistance.

However, current research mainly focuses on exogenous Spm and Spd, while the specific mechanisms of exogenous Put on wheat’s physiological and biochemical processes remain underexplored. Furthermore, differences in physiological responses among wheat varieties with different Cd tolerances remain an area for further research. Thus, this study hypothesizes that exogenous Put alleviates Cd stress in wheat through antioxidant activation and physiological modulation, with distinct responses between Cd-tolerant and Cd-sensitive cultivars. To test this, two wheat varieties with contrasting Cd tolerance were selected to investigate the physiological and biochemical effects of Put treatment on Cd tolerance in wheat varieties with differing sensitivities. This study will help provide deeper insights into how Put modulates wheat responses under Cd stress. The findings offer a physiological regulatory basis for breeding Cd-tolerant wheat varieties and optimizing precision application protocols for growth regulators in Cd-contaminated areas.

## 2 Materials and methods

### 2.1 Experimental materials and reagents

Building on our previous research (Zhong et al., 2024), we selected two wheat varieties as experimental materials: Cd-sensitive Chang 6475 and Cd-tolerant Changmai 4013. Both varieties were developed by the Wheat Research Laboratory at Shanxi Agricultural University’s Millet Institute. Changmai 4013 (parental cross: Jingken 49/Changmai 6673) was approved for irrigated areas in central Shanxi Province. Chang 6475 was derived from the cross between parental lines 13–5926 and 12-11624. Cd^2+^ was supplied as cadmium chloride (CdCl_2_·2.5H_2_O), and Put, also known as 1,4–diaminobutane, was sourced from Shanghai Macklin Biochemical Technology Co., Ltd. (China). The concentrations of the Cd solution and Put solution were determined based on previous research conducted by the research group. According to the findings of (Zhong et al., 2024), an 80 mg·L^−1^ Cd solution and Put solutions of 0.05, 0.1, 0.2, 0.4, 0.8, and 1.6 mM were selected for the experiment.

### 2.2 Experimental design

After seed sterilization, the seeds were soaked in sterile beakers containing 0, 0.05, 0.1, 0.2, 0.4, 0.8, or 1.6 mM Put solution at 28°C for 2 h. After air-drying on a sterile bench, the treated seeds were placed in Petri dishes and cultured with 5 mL Cd solution. For the control treatment (CK), 5 mL distilled water was used instead. The treatments were as follows: (1) CK: distilled water; (2) T0: Cd + 0 mM Put; (3) T1: Cd + 0.05 mM Put; (4) T2: Cd + 0.1 mM Put; (5) T3: Cd + 0.2 mM Put; (6) T4: Cd + 0.4 mM Put; (7) T5: Cd + 0.8 mM Put; (8) T6: Cd + 1.6 mM Put. In each treatment, 50 soaked wheat seeds were sown on double layers of filter paper in 90 mm Petri dishes (Whatman No. 1). The Petri dishes were placed in an artificial climate chamber set at (25 ± 1)°C and 40% relative humidity (BIC-300, 2020). Five replicates of each treatment were supplemented with Cd solution every 2 day. The experiment ended on the eighth day. Seeds were counted as germinated when the germ length grew to more than 1/2 of the seed diameter. The number of germination was recorded once a day at a fixed time during the incubation period and germination parameters were calculated (Equations 1-6) at the end of incubation (Mei et al., 2012). In this study, “treatment” specifically refers to the experimental procedure of soaking seeds in Put solutions at different concentrations.


(1) $$\begin{array}{c} Germination\;energy\left(GE\right)\\ =\frac{Number\;of\;seeds\;germinated\;on\;day\;3}{Total\;number\;of\;seeds\times 100\%} \end{array}$$

(2) $$\begin{array}{c} Germination\;percentage\left(GP\right)\\ =\frac{Number\;of\;seeds\;germinated\;on\;day\;7}{Total\;number\;of\;seeds\times 100\%} \end{array}$$

(3) $$\begin{array}{c} Germination\;index\left(GI\right)=1.00\times n{\text{d}}_{1}+0.75\times n{\text{d}}_{2}+0.50\\ \times n{\text{d}}_{3}+0.25\times n{\text{d}}_{4}(\text{where}\;n{\text{d}}_{1},n{\text{d}}_{2},n{\text{d}}_{3},\;\text{and}\;n{\text{d}}_{4}\;\text{are}\;\\ \text{the}\;\text{germination}\;\text{rates}\;\text{on}\;\text{days}\;1,\;2,\;3,\;\text{and}\\ \;4,\;\text{respectively}) \end{array}$$

(4) $$\begin{array}{c} Vigor\;index\left(VI\right)\\ =Germination\;index\;\times Average\;length\;of\;sprouts\;on\;day\;8 \end{array}$$

(5) $$\begin{array}{c} Germination\;Cd\;resistance\;index\left(GCRI\right)\\ =\frac{Germination\;index\;under\;Cd\;stress}{Control\;germination\;index} \end{array}$$

(6) $$\begin{array}{c} Vigor\;Cd\;resistance\;index\left(VCRI\right)\\ =\frac{Vigor\;index\;under\;Cd\;stress}{Control\;vigor\;index} \end{array}$$

### 2.3 Morphological observation and growth parameter measurement

After 8 days, 10 randomly selected plants with consistent growth were measured for growth parameters (Ji et al., 2022). Root length and bud length were determined utilizing a straightedge, and their respective fresh weights (FW) were weighed utilizing an electronic balance, then the samples were dried until constant mass and weighed for dry weight (DW), and the ratio of the dry mass of the aboveground and belowground parts of the plant was the root-to-shoot ratio (R/S).

For each treatment, 5 fresh wheat roots were randomly collected and washed with distilled water. Fresh roots were scanned using a scanner (Epson Perfection V800 PHOTO, Epson, Japan) and analyzed using WinRHIZO software for root parameters.

### 2.4 Determination of Cd content

Approximately 0.1-0.4 g of dried rhizome tissue samples were weighed and subjected to analysis. The Cd content was determined using inductively coupled plasma mass spectrometry (ICP-MS) in accordance with Chinese National Standard GB 5009.268-2016. Detailed procedures are provided in **Supplementary Table S1**. The calculation formula is as follows (Equation 7):


(7) $$Elementcontent\left(mg/kg\right)=\frac{c\times V}{m}\times D$$

In the formula: c represents the concentration of the element in the solution (mg/L); V represents the extraction volume (mL); D represents the dilution factor; m represents the sample mass (g).

### 2.5 Determination of osmotic regulation and membrane lipid peroxidation indicators

Evans blue staining was employed to assess bud damage, as described by Das et al. (2025). For soluble protein (SP) content as described by Zou (2000), it was determined by using the Coomassie Brilliant Blue method. Free proline (Pro) content was determined as described by Li et al. (2021) using the acid ninhydrin assay. The malondialdehyde (MDA) content was assessed using the thiobarbituric acid method, as described by Zou (2000). Relative water content and relative electrical conductivity were quantified based on the methods described by Polash et al. (2018) and (Lutts and Guerrier, 1995), respectively. 0.1 g of fresh buds were weighed and sectioned, and after soaking in distilled water for 12 h, the extract was measured using a conductivity meter to record the initial conductivity value EC1; subsequently, it was boiled for 15 min in a thermostatic water bath, cooled and shaken, and the conductivity value EC2 was measured again. A suitable amount of shoot material from each treatment was randomly selected, cut into segments, and weighed to determine its FW. The saturated mass (TW) was then determined after 12 h of immersion in distilled water; finally, the samples were dried and their DW was recorded. The calculation formulas for the relevant parameters are as follows (Equations 8, 9):


(8) Relative electrical conductivity(REL)=EC1/EC2×100%

(9) $$Relative\;water\;content\left(RWC\right)=\frac{\left(FW-DW\right)}{\left(TW-DW\right)}\times 100\%$$

### 2.6 Determination of ROS, antioxidant enzymes, and non-enzymatic antioxidants

Determination of O_2_·^–^ content using the hydroxylamine method (Kour et al., 2024; Elstner and Heupel, 1976). Determination of H_2_O_2_ content using acetone extraction method (Han et al., 2025). Determination of ·OH content using 2-deoxyribose method (Liu et al., 2018; Halliwell et al., 1987). The activities of superoxide dismutase (SOD) and catalase (CAT) were measured using the nitroblue tetrazolium photoreduction method and ultraviolet spectrophotometry, respectively (Wu et al., 2014). Peroxidase (POD) activity was quantified using the guaiacol colorimetric technique (Wang et al., 2012). Glutathione reductase (GR) activity was measured based on the method described by Kour et al. (2024) and Foyer and Halliwell (1976). Ascorbate peroxidase (APX) activity was measured according to the method of Nakano and Asada (Xu et al., 2024; Nakano and Asada, 1981). Ascorbic acid (AsA) and dehydroascorbic acid (DHA) contents were measured with reference to the method of Jiang and Zhang (2001). The levels of reduced glutathione (GSH) and oxidized glutathione (GSSG) were determined using the method described by Nagalakshmi and Prasad (Nagalakshmi and Prasad, 2001). Detailed procedures are provided in **Supplementary Table S1**.

### 2.7 Determination of endogenous PA content

Endogenous PAs (Spd, Spm, Put) were quantified using liquid chromatography-mass spectrometry (LC-MS) in positive ion mode (Pál et al., 2022). The instrumentation and reagents used are listed in **Table 1**. Fresh seedling tissue samples were finely chopped, and 50 mg aliquots were weighed. Each aliquot was combined with 500 µL of a methanol, acetonitrile mixture (1:1, v/v), vortexed for 60 s, and then homogenized using a grinder (18 cycles). The mixture was subsequently sonicated for 10 min and centrifuged at 17000g for 15 min. The resulting supernatant was collected for analysis.

**Table 1:** Instruments and reagents.

Instruments and reagents	Producer
High-Class Liquid Chromatography system (HCLASS)	Waters
Triple quadrupole mass spectrometer (AB 4500)	AB Sciex
Chromatographic column (BEH C18 1.7 µm, 2.1 × 100 mm)	Waters
Methanol	Merck (Darmstadt, Germany)
Ultrapure water	Merck (Darmstadt, Germany)
Acetonitrile	Merck (Darmstadt, Germany)
Formic acid	Shanghai Macklin Biochemical Co., Ltd

A working internal standard (IS) solution was prepared by diluting the IS stock in 75% methanol/water. Calibration standards were prepared by spiking the mixed standard solution with the IS working solution at a 1:1 volume ratio. The IS working solution itself was diluted 1:1 with 75% methanol/water to create the calibration dilution solution, which was then serially diluted to specific concentrations. The final IS concentration in the prepared solutions was 250 ng/mL.

The LC separation was performed using the following conditions: column temperature, 40°C; injection volume, 1 µL; mobile phase A, 0.1% formic acid in water; mobile phase B, acetonitrile; ionization mode, positive. The gradient elution program is detailed in **Table 2**.

**Table 2:** Mobile phase gradient elution program.

Time (min)	Flow-rate (µL/min)	A%	B%
0.00	400	95	5
1.50	400	95	5

Mass spectrometric detection was carried out on an AB Sciex 4500 system operating in multiple reaction monitoring (MRM) mode with negative polarity for data acquisition. Electrospray ionization (ESI) source parameters were optimized as follows: source temperature (Gas Temp), 500°C; curtain gas (CUR), 25 psi; collision gas (CAD), 10 psi; ion spray voltage (IS), 4500V; and nebulizer gas temperature (TEM), 500°C. Ion pair information can be found in **Supplementary Table S2**. Quantification was performed using the internal standard method, whereby calibration curves were constructed by plotting the peak area ratios (analyte/internal standard) against corresponding standard concentrations. Sample concentrations were then calculated based on these curves, and the PA content was determined accordingly.

### 2.8 Comprehensive evaluation and statistical analysis

Each treatment was replicated five times, and data were analyzed using IBM SPSS Statistics (Version 27.0.1; IBM SPSS Inc., USA), with a one-way analysis of variance applied to all datasets. Results are expressed as means ± standard deviation (Jin et al., 2020). Graphs were generated using Origin 2022 and R software (version 4.3.2). Raw mass spectrometry data were processed using MultiQuant software (version 3.0.3). The optimal application rate of Put was determined using the membership function average (MV) evaluation method. A higher MV indicates a more effective treatment concentration (Equations 10-12) (Shen et al., 2024).


(10) $$V_{ija}=\frac{\left(X_{ij}-X_{jmin}\right)}{\left(X_{jmax}-X_{jmin}\right)}$$

(11) $$V_{ijb}=1-\frac{\left(X_{ij}-X_{jmin}\right)}{\left(X_{jmax}-X_{jmin}\right)}$$

(12) $$MV_{i}=\frac{1}{n}\sum_{j=1}^{n}V_{ij}$$

Where, *V_ij_*: represents the value of the affiliation function of indicator j under different Put treatments; *V_ija_* and *V_ijb_*: represent the positive and negative correlation with the treatments; *X_ij_*: represents the measured mean value of indicator *j* under different Put treatments; *X_j_*_max_ and *X_j_*_min_: represent the maximum and minimum values of indicator *j*; *MV_i_*: the mean value of the affiliation function of all indicators under different Put treatments.

## 3 Results

### 3.1 Wheat seed germination

Under different concentrations of Put treatment (T0–T6), no statistically significant effects were observed on germination energy (GE), germination percentage (GP), germination index (GI), or germination Cd resistance index (GCRI) in both wheat varieties under Cd stress (*P* > 0.05) (**Table 3**). Compared with CK, the GCRI of Changmai 4013 in T0 increased significantly by 40%, but its vigor Cd resistance index (VCRI) decreased significantly by 40.00% (*P<* 0.05). For Chang 6475, GE and GI in T0 increased significantly by 30.40% and 39.77%, respectively, while its Vigor index (VI) decreased significantly by 40.29% (*P<* 0.05). Under different Put treatments, for Changmai 4013, VI and VCRI peaked at T2 and T3, showing significant increases of 19.14% and 38.33% over T0, respectively (*P<* 0.05). In contrast, Chang 6475 reached its VI and VCRI peaks at T3 and T2, with 38.55% and 18.95% increases relative to T0, respectively (*P<* 0.05). These results demonstrate that exogenous Put seed soaking significantly enhances VI and VCRI in both varieties.

**Table 3:** The impact of Put on germination parameters of Changmai 4013 and Chang 6475 under Cd stress.

Variety	Treatment	Germination energy (GE)/%	Germination percentage (GP)/%	Germination index (GI)	Vigor index (VI)	Germination on Cd resistance index (GCRI)	Vigor Cd resistance index (VCRI)
Changmai 4013	CK	86.00 ± 6.00a	90.67 ± 3.06b	1.36 ± 0.07a	6.40 ± 0.27b	1.00 ± 0.00b	1.00 ± 0.00a
	T0	89.33 ± 6.11a	94.00 ± 4.00ab	1.28 ± 0.10a	6.06 ± 0.87bc	1.40 ± 0.07a	0.60 ± 0.06c
	T1	90.67 ± 6.11a	94.67 ± 6.11ab	1.28 ± 0.13a	6.31 ± 0.43b	1.42 ± 0.05a	0.73 ± 0.03b
	T2	94.00 ± 4.00a	98.00 ± 2.00a	1.35 ± 0.08a	7.22 ± 0.09a	1.52 ± 0.15a	0.82 ± 0.12b
	T3	92.67 ± 2.31a	96.67 ± 1.15ab	1.29 ± 0.09a	6.22 ± 0.41b	1.44 ± 0.12a	0.83 ± 0.11b
	T4	92.00 ± 3.46a	96.00 ± 2.00ab	1.26 ± 0.04a	6.07 ± 0.23bc	1.52 ± 0.07a	0.79 ± 0.02b
	T5	90.00 ± 3.46a	96.00 ± 2.00ab	1.24 ± 0.05a	5.90 ± 0.36bc	1.47 ± 0.04a	0.75 ± 0.03b
	T6	90.67 ± 6.43a	93.33 ± 4.16ab	1.25 ± 0.09a	5.28 ± 0.31c	1.50 ± 0.11a	0.74 ± 0.07b
Chang 6475	CK	68.00 ± 2.00b	93.33 ± 4.16a	0.88 ± 0.06b	8.34 ± 0.51a	1.00 ± 0.00a	1.00 ± 0.00b
	T0	88.67 ± 6.11a	96.00 ± 2.00a	1.23 ± 0.06a	4.98 ± 0.49c	0.94 ± 0.07a	0.95 ± 0.14b
	T1	90.00 ± 5.29a	96.00 ± 2.00a	1.25 ± 0.05a	6.08 ± 0.25b	0.94 ± 0.09a	0.99 ± 0.07b
	T2	94.67 ± 5.77a	98.00 ± 0.00a	1.34 ± 0.13a	6.86 ± 1.00b	0.99 ± 0.06a	1.13 ± 0.01a
	T3	90.00 ± 3.46a	92.67 ± 4.62a	1.27 ± 0.11a	6.90 ± 0.93b	0.95 ± 0.06a	0.97 ± 0.06b
	T4	92.00 ± 2.00a	94.67 ± 3.06a	1.34 ± 0.06a	6.60 ± 0.20b	0.93 ± 0.03a	0.95 ± 0.04b
	T5	91.33 ± 3.06a	94.00 ± 3.46a	1.29 ± 0.04a	6.25 ± 0.22b	0.91 ± 0.04a	0.92 ± 0.06bc
	T6	92.00 ± 5.29a	95.33 ± 4.62a	1.32 ± 0.10a	6.14 ± 0.56b	0.92 ± 0.07a	0.82 ± 0.05c

Bar bars with different lowercase letters are significantly different by Duncan’s test (*p*< 0.05). Values are expressed as the mean ± SD of five replicates.

### 3.2 Wheat seedling growth

**Figure 1:**
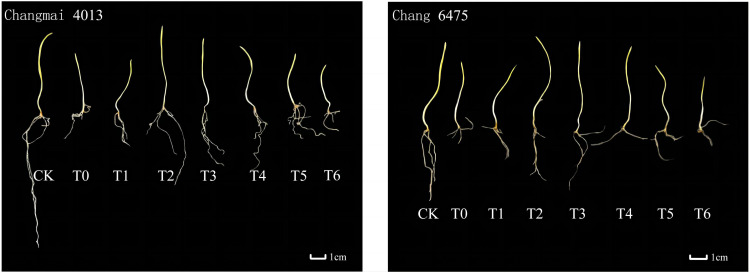
The impact of Put on plant growth of Changmai 4013 and Chang 6475 under Cd stress.

Under the T0 treatment, the growth of both wheat varieties was poorer compared to the CK group. However, Put treatment significantly ameliorated this growth inhibition (**Figure 1**). T0 treatment significantly (*P<* 0.05) reduced the fresh weight (FW) of Changmai 4013 by 2.74% (**Table 4**), compared to CK. Under T0, the root length (RL) and bud length (BL) of Chang 6475 were significantly (*P<* 0.05) lower than those of CK, with reductions of 26.03% and 36.83%, respectively. Different concentrations of Put seed soaking significantly affected the BL, FW, dry weight (DW), and root-to-shoot ratio (R/S) of the two wheat varieties under Cd treatment (T0). For Changmai 4013, the RL, BL, and DW reached their maximum at the T2 treatment, with increases of 27.62%, 13.77%, and 8.33%, respectively, compared to T0. The FW and R/S reached their peak at T3, with significant increases of 18.31% and 32.22%, respectively, compared to T0 (*P<* 0.05). For Chang 6475, the maximum BL, FW, and DW were observed at T3, with increases of 34.49%, 13.51%, and 7.69%, respectively, compared to T0. Therefore, T2 and T3 treatments elicited positive responses in seedling growth and biomass accumulation for both wheat varieties. Notably, exogenous Put seed soaking demonstrated a more pronounced enhancement in shoot elongation and biomass production in Chang 6475 compared to Changmai 4013.

**Table 4:** The impact of Put on seedling growth of Changmai 4013 and Chang 6475 under Cd stress.

Variety	Treatment	Root length (RL)/cm	Bud length (BL)/cm	Fresh weight (FW)/g	Dry weight (DW)/g	Root-to-shoot ratio (R/S)
Changmai 4013	CK	5.59 ± 0.24c	4.72 ± 0.14b	0.73 ± 0.02d	0.12 ± 0.00ab	0.94 ± 0.01d
	T0	5.25 ± 0.06c	4.72 ± 0.34b	0.71 ± 0.01e	0.12 ± 0.01bc	0.90 ± 0.02d
	T1	5.39 ± 0.12c	4.95 ± 0.17b	0.73 ± 0.00d	0.13 ± 0.01ab	0.90 ± 0.04d
	T2	6.70 ± 0.27a	5.37 ± 0.23a	0.82 ± 0.01b	0.13 ± 0.01a	1.08 ± 0.04c
	T3	6.06 ± 0.11b	4.84 ± 0.06b	0.84 ± 0.02a	0.13 ± 0.01ab	1.19 ± 0.03a
	T4	5.27 ± 0.17c	4.81 ± 0.02b	0.81 ± 0.01b	0.13 ± 0.01ab	1.15 ± 0.02ab
	T5	5.25 ± 0.43c	4.77 ± 0.25b	0.77 ± 0.01c	0.12 ± 0.01bc	1.09 ± 0.07bc
	T6	4.49 ± 0.34d	4.24 ± 0.09c	0.71 ± 0.02de	0.11 ± 0.00c	1.06 ± 0.02c
Chang 6475	CK	6.07 ± 0.17b	6.38 ± 0.26a	0.78 ± 0.01b	0.13 ± 0.00ab	0.98 ± 0.00ab
	T0	4.49 ± 0.29a	4.03 ± 0.23e	0.74 ± 0.02b	0.13 ± 0.00c	0.97 ± 0.03ab
	T1	4.97 ± 0.48a	4.85 ± 0.14cd	0.75 ± 0.01b	0.13 ± 0.00bc	0.99 ± 0.04ab
	T2	5.19 ± 0.50a	5.13 ± 0.29bc	0.78 ± 0.02b	0.13 ± 0.00ab	0.97 ± 0.03ab
	T3	5.24 ± 0.42a	5.42 ± 0.27b	0.84 ± 0.08a	0.14 ± 0.00a	1.01 ± 0.12a
	T4	5.18 ± 0.08a	4.92 ± 0.18cd	0.76 ± 0.00b	0.12 ± 0.00cd	0.91 ± 0.04b
	T5	5.11 ± 0.17a	4.83 ± 0.07cd	0.73 ± 0.01b	0.12 ± 0.00d	0.90 ± 0.03b
	T6	4.89 ± 0.64a	4.66 ± 0.09c	0.65 ± 0.00c	0.11 ± 0.00e	0.90 ± 0.03b

Bar bars with different lowercase letters are significantly different by Duncan’s test (*p*< 0.05). Values are expressed as the mean ± SD of five replicates.

### 3.3 Root morphological parameters

**Figure 2:**
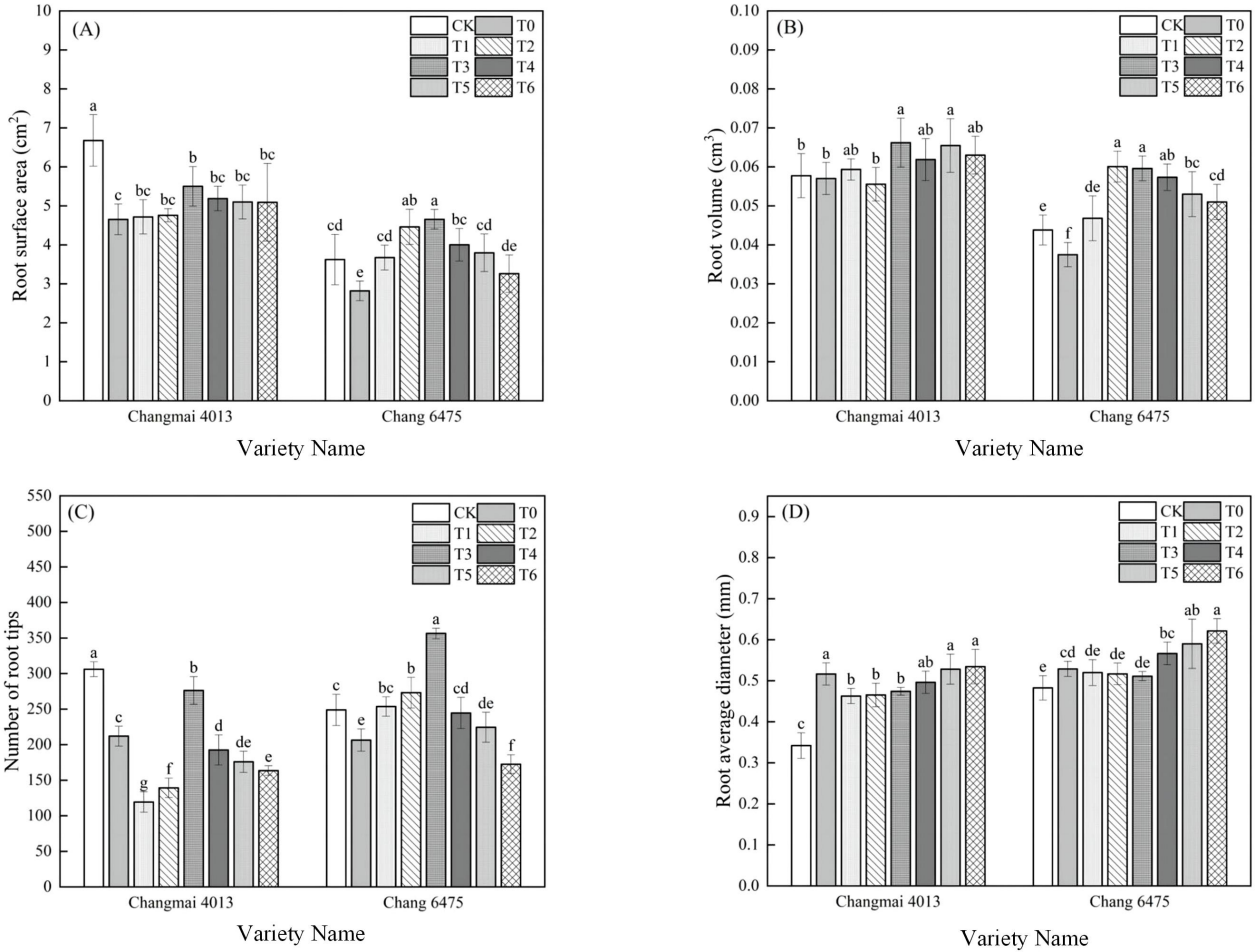
The impact of Put on **(A)** root surface area (RSA), **(B)** root volume (RV), **(C)** number of root tips (RT), **(D)** root average diameter (RAD) of Changmai 4013 and Chang 6475 under Cd stress. CK: Distilled water; T0: Cd + 0 mM Put; T1: Cd + 0.05 mM Put; T2: Cd + 0.1 mM Put; T3: Cd + 0.2 mM Put; T4: Cd + 0.4 mM Put; T5: Cd + 0.8 mM Put; T6: Cd + 1.6 mM Put. Bar bars with different lowercase letters are significantly different by Duncan’s test (*p<* 0.05). Data are presented as the mean of five independent mean ± SD.

Compared to CK, the root surface area (RSA) and the number of root tips (RT) in Changmai 4013 decreased significantly (*P<* 0.05) by 30.39% and 30.75%, respectively, and the root average diameter (RAD) increased significantly (*P<* 0.05) by 52.94% under Cd treatment (T0); In Chang 6475, the RSA, RV, and RT decreased significantly (*P<* 0.05) by 22.10%, 15.91%, and 17.04%, respectively, and the RAD increased significantly (*P<* 0.05) by 10.42% (**Figure 2**). Under T0 treatment, seed soaking with Put promoted root development in both wheat varieties. In Changmai 4013, the RSA, root volume (RV), and RT increased significantly (*P<* 0.05) by 18.28%, 16.14%, and 30.28%, respectively, under the T3 treatment. For Chang 6475, the RSA and RT increased significantly (*P<* 0.05) by 64.89% and 72.65%, respectively. Exogenous Put seed soaking proved more effective in improving the root system of Chang 6475, compared to Changmai 4013.

### 3.4 Cd accumulation

**Figure 3:**
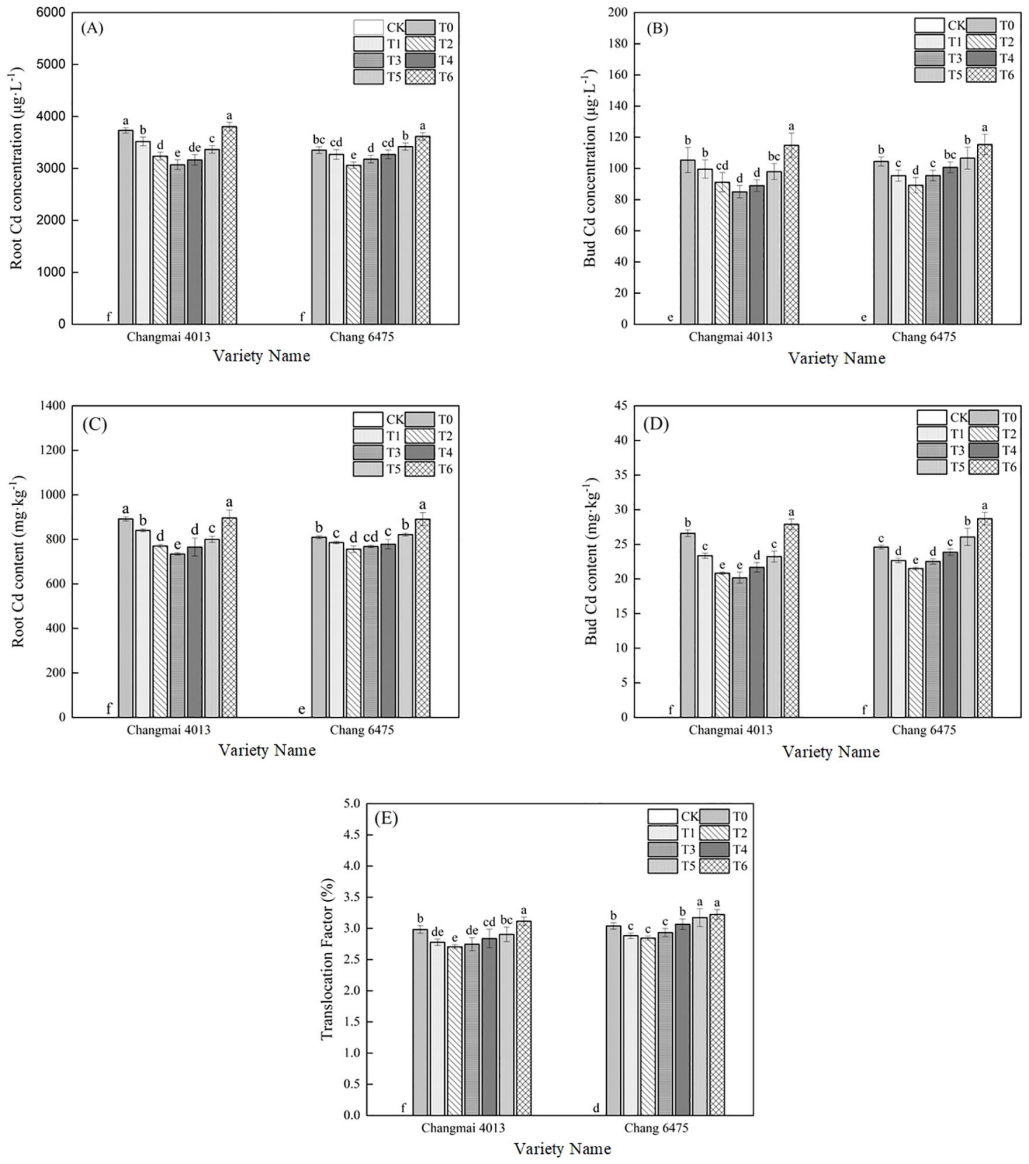
Correlation analysis of Put treatments on Chang 6475 under Cd stress. Blue color indicates positive correlation, red color indicates negative correlation, and sector area indicates the magnitude of the correlation coefficient. GE, germination energy; GP, germination percentage; GI, germination index; VI, vigor index; GCRI, germination Cd resistance index; VCRI, vigor Cd resistance index; RL, root length; BL, bud length; FW, fresh weight; DW, dry weight; R/S, root-to-shoot ratio; RWC, relative water content; REL, relative electrical conductivity; RSA, root surface area; RV, root volume; RT, number of root tips; RAD, root average diameter; BCC, bud Cd concentration; RCC, root Cd concentration; BCT, bud Cd content; RCT, root Cd content; TF, translocation factor; SOD, superoxide dismutase activity; POD, peroxidase activity; CAT, catalase activity; APX, ascorbate peroxidase activity; GR, glutathione reductase activity; SP, soluble protein; O_2_·^–^, superoxide anion rate; MDA, malondialdehyde; H_2_O_2_, hydrogen peroxide; Pro, free proline; ·OH, hydroxyl radical; GSSG, oxidized glutathione; GSH, reduced glutathione; AsA, ascorbic acid; DHA, dehydroascorbic acid; Spd, spermidine; Spm, spermine; Put, putrescine.

Compared to T0, both varieties exhibited a trend of initial decrease followed by increase in bud Cd concentration (BCC), root Cd concentration (RCC), bud Cd content (BCT), root Cd content (RCT), and translocation factor (TF) with rising Put concentrations. (**Figure 3**) For variety Changmai 4013, the minimum values of BCC, RCC, BCT, and RCT were observed under T3 treatment, showing significant (*P<* 0.05) reductions of 19.35%, 17.75%, 24.19%, and 17.65% compared to T0, respectively. Its TF reached the lowest value at T2 treatment, decreasing by 9.40% compared to T0. In contrast, for variety Chang 6475, all parameters (BCC, RCC, BCT, RCT, and TF) attained minimum values at T2 treatment, with significant (*P<* 0.05) reductions of 14.61%, 8.72%, 12.57%, 6.60%, and 6.58% relative to T0, respectively. These results indicate that exogenous Put effectively reduced Cd accumulation in both roots and bods of the two wheat varieties.

### 3.5 Oxidative stress

**Figure 4:**
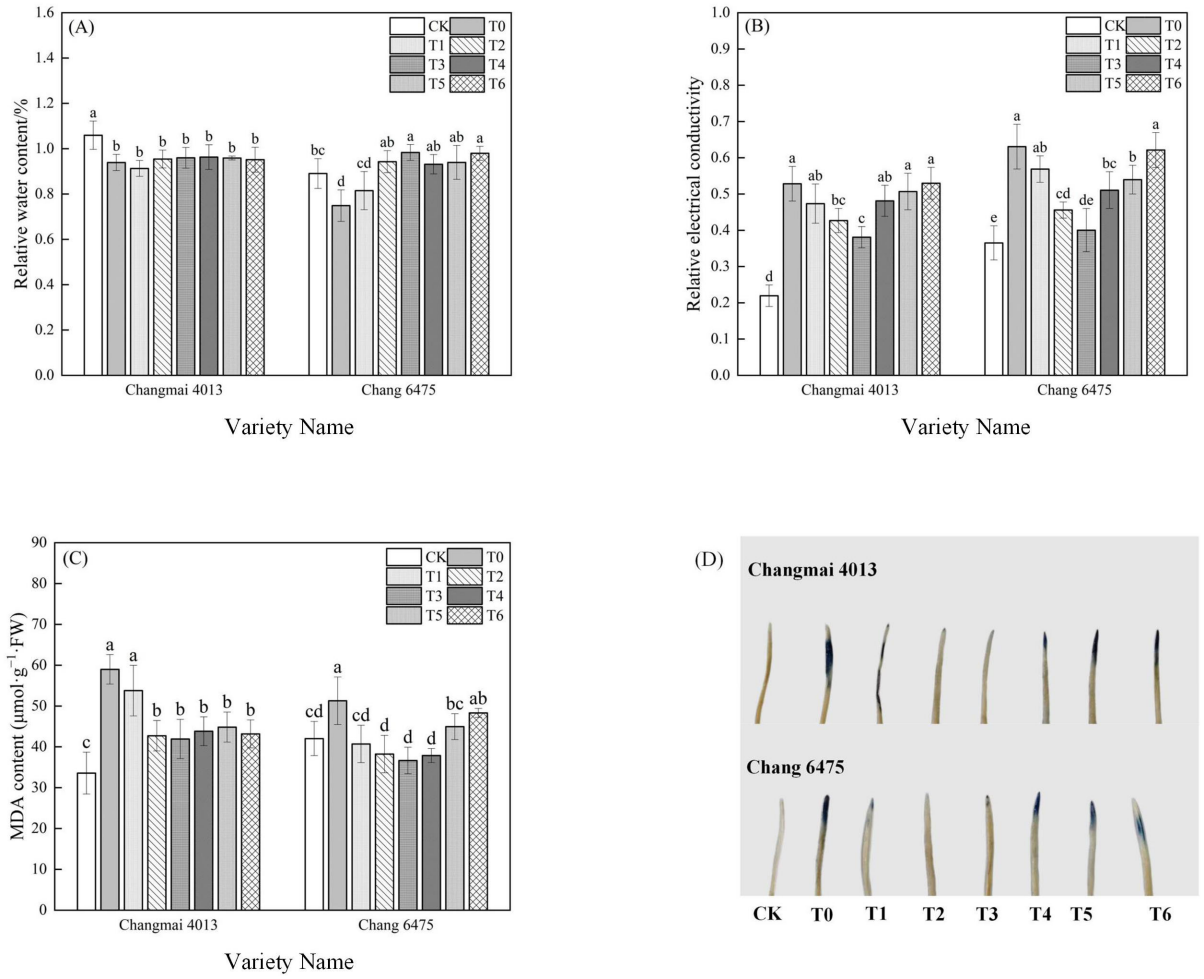
The impact of Put on **(A)** relative water content (RWC), **(B)** relative electrical conductivity (REL), **(C)** malondialdehyde (MDA), **(D)** embryo cell vitality of Changmai 4013 and Chang 6475 under Cd stress. CK: Distilled water; T0: Cd + 0 mM Put; T1: Cd + 0.05 mM Put; T2: Cd + 0.1 mM Put; T3: Cd + 0.2 mM Put; T4: Cd + 0.4 mM Put; T5: Cd + 0.8 mM Put; T6: Cd + 1.6 mM Put. Bar bars with different lowercase letters are significantly different by Duncan’s test (*p*< 0.05). Data are presented as the mean of five independent mean ± SD.

Cd treatment (T0) significantly (*P<* 0.05) decreased the relative water content (RWC) of Changmai 4013 by 11.32%, and of Chang 6475 by 15.73% compared to CK (**Figure 4A**). However, Put treatment improved RWC, with a 30.67% increase observed in Chang 6475 under T3.

Relative to CK, Cd treatment (T0) significantly increased the relative electrical conductivity (REL) and malondialdehyde (MDA) content in both varieties (**Figure 4B, C**). Under the various Put concentrations, the REL and MDA content of Changmai 4013 and Chang 6475 were minimized under T3 treatment. Specifically, compared to T0, the REL and MDA content of Changmai 4013 decreased significantly (*P<* 0.05) by 28.30% and 28.94%, respectively; for Chang 6475, the reductions were 36.51% and 28.54%, respectively.

Embryo staining was performed using Evans blue dye, with blue spots indicating cell death. A larger stained area correlates with a lower cell survival rate (**Figure 4D**). Compared to CK, the formation of blue spots in embryos under Cd stress (T0) was significantly greater, reflecting the stress induced by Cd. Under the Put treatments (T1-T6), the stained area of the germ showed a tendency to fall and then to rise, with the least damage observed in embryos treated with T2 treatments in both varieties. These results suggest that certain concentrations of Put treatment can provide protection to wheat seedlings from oxidative damage induced by Cd stress, and the protective effect was more significant for the highly sensitive variety Chang 6475.

### 3.6 SP and Pro content

**Figure 5:**
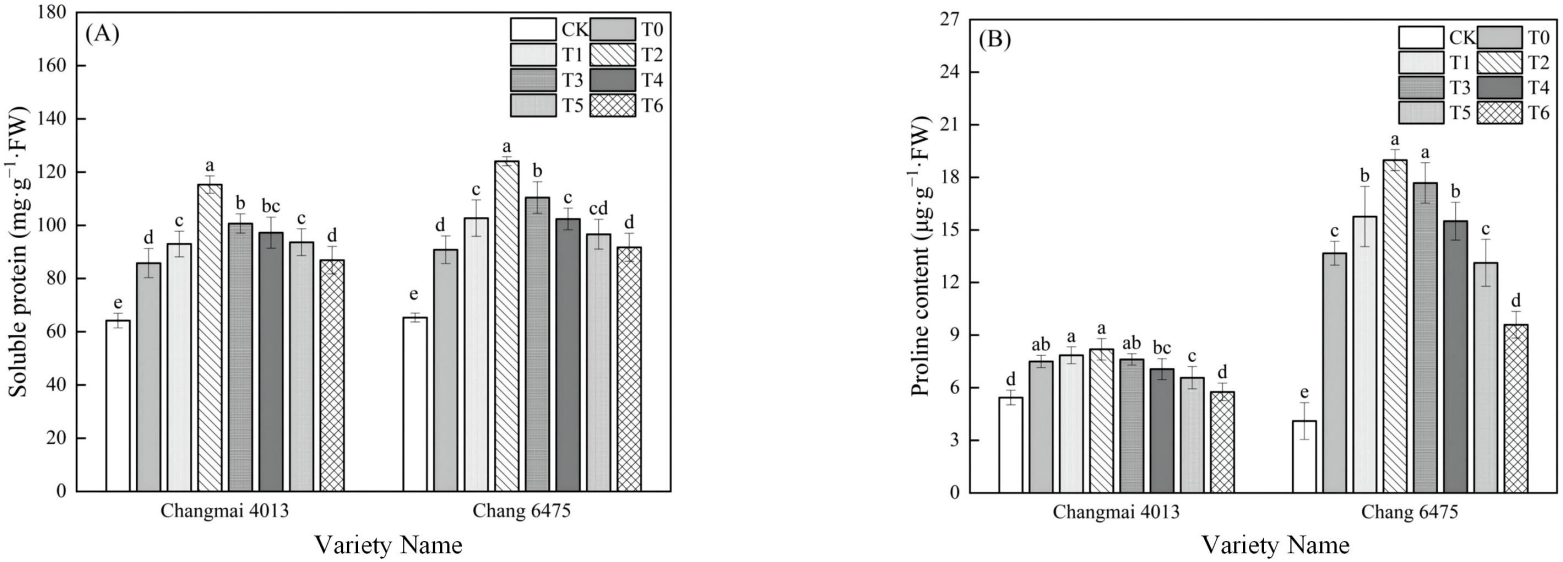
The impact of Put on **(A)** soluble protein (SP), **(B)** free proline (Pro) of Changmai 4013 and Chang 6475 under Cd stress. CK: Distilled water; T0: Cd + 0 mM Put; T1: Cd + 0.05 mM Put; T2: Cd + 0.1 mM Put; T3: Cd + 0.2 mM Put; T4: Cd + 0.4 mM Put; T5: Cd + 0.8 mM Put; T6: Cd + 1.6 mM Put. Bar bars with different lowercase letters are significantly different by Duncan’s test (*p<* 0.05). Data are presented as the mean of five independent mean ± SD.

Cd stress (T0) significantly increased the SP and Pro contents of Changmai 4013 by 33.64% and 37.87%, and that of Chang 6475 by 38.99% and 233.41% compared with CK (*P<* 0.05) (**Figure 5**). Under Cd stress (T0), the SP content of Changmai 4013 was significantly increased by 34.39% in T2 treatment; the Pro and SP content of Chang 6475 were both significantly increased by 38.84% and 36.61% in T2 treatment (*P<* 0.05). Notably, after Put treatment, both the SP and Pro contents in Chang 6475 were higher than those in Changmai 4013, indicating that exogenous Put had a more pronounced alleviating effect on Chang 6475.

### 3.7 O_2_·^–^, H_2_O_2_ and ·OH content

**Figure 6:**
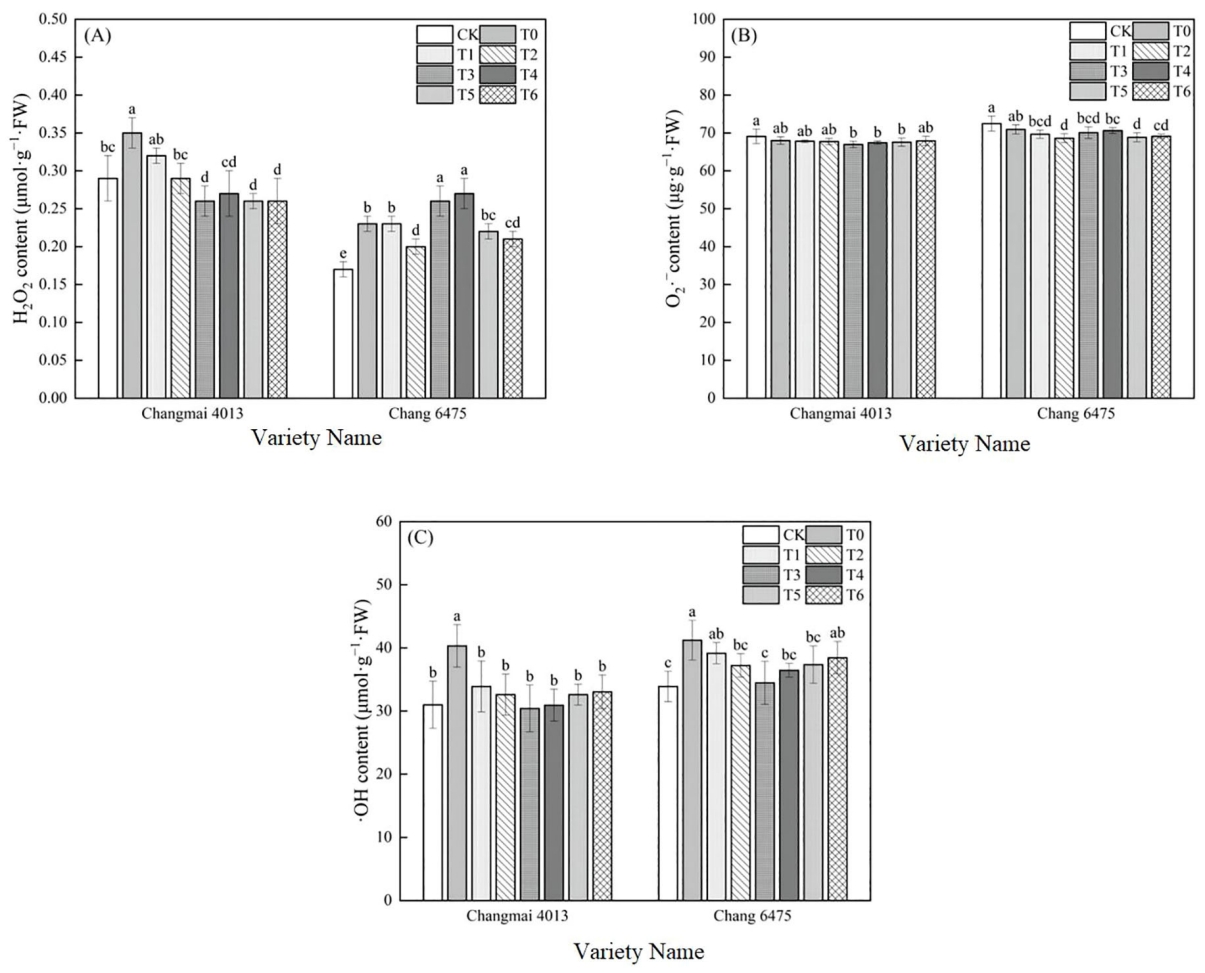
The impact of Put on **(A)** hydrogen peroxide (H_2_O_2_), **(B)** superoxide anion rate (O_2_·^–^), **(C)** hydroxyl radical (·OH) of Changmai 4013 and Chang 6475 under Cd stress. CK: Distilled water; T0: Cd + 0 mM Put; T1: Cd + 0.05 mM Put; T2: Cd + 0.1 mM Put; T3: Cd + 0.2 mM Put; T4: Cd + 0.4 mM Put; T5: Cd + 0.8 mM Put; T6: Cd + 1.6 mM Put. Bar bars with different lowercase letters are significantly different by Duncan’s test (*p<* 0.05). Data are presented as the mean of five independent mean ± SD.

Compared to CK, T0 treatment significantly increased H_2_O_2_ and ·OH levels in both Changmai 4013 and Chang 6475, reaching statistically significant differences (**Figure 6**). Under different Put treatments, H_2_O_2_ and ·OH levels in Changmai 4013 were minimized under T3 treatment, showing significant (*P<* 0.05) reductions of 25.71% and 24.56%, respectively, compared to T0. In Chang 6475, the lowest H_2_O_2_ and O_2_·^–^ levels were observed under T2 treatment, with significant (*P<* 0.05) decreases of 13.04% and 3.24%, respectively, while ·OH levels were minimized under T3 treatment, showing a significant (*P<* 0.05) reduction of 16.38%. These findings suggest that Put plays a key role in maintaining the integrity of cell membranes.

### 3.8 Antioxidant metabolism

**Figure 7:**
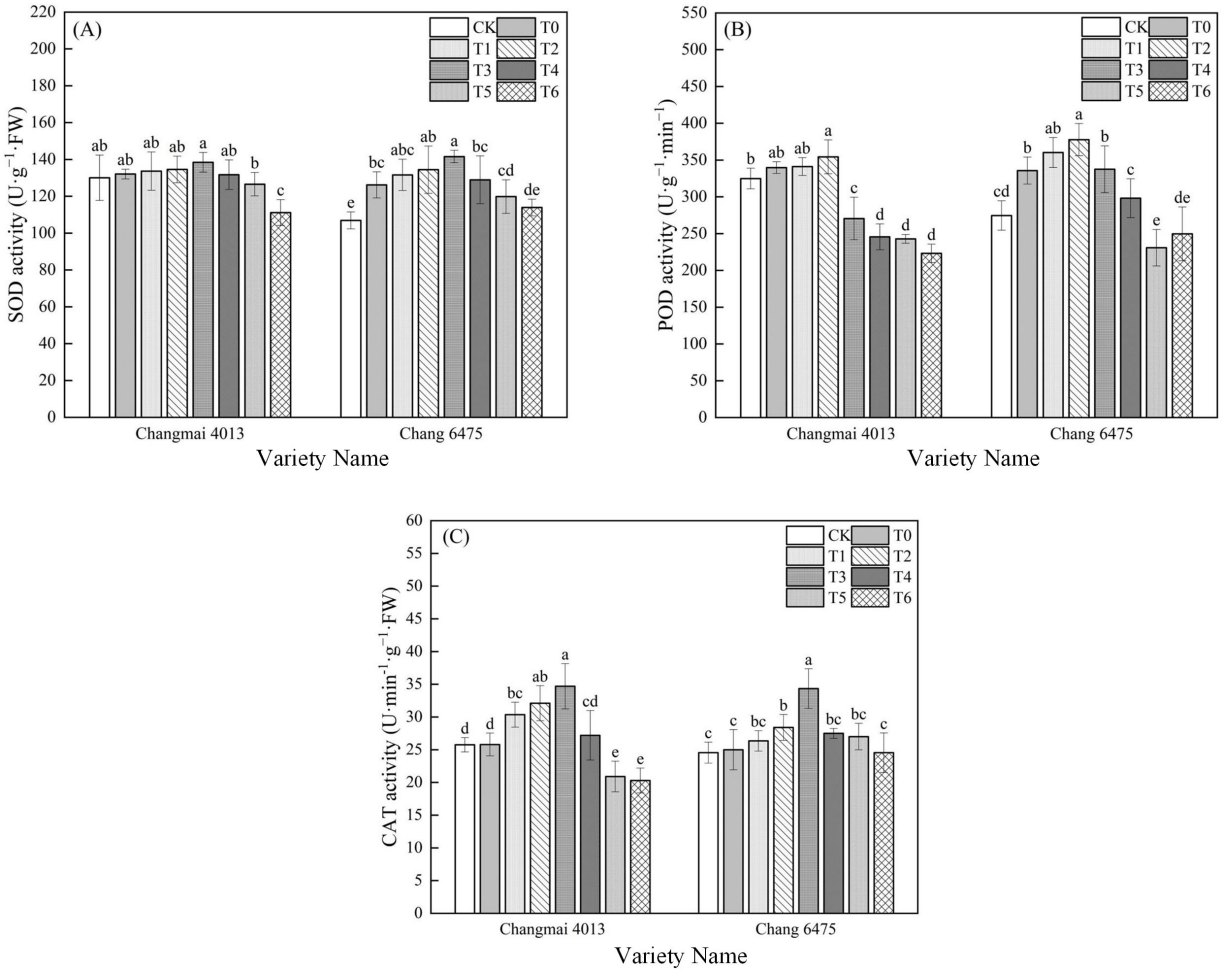
The impact of Put on **(A)** superoxide dismutase activity (SOD), **(B)** peroxidase activity (POD), **(C)** catalase activity (CAT) of Changmai 4013 and Chang 6475 under Cd stress. CK: Distilled water; T0: Cd + 0 mM Put; T1: Cd + 0.05 mM Put; T2: Cd + 0.1 mM Put; T3: Cd + 0.2 mM Put; T4: Cd + 0.4 mM Put; T5: Cd + 0.8 mM Put; T6: Cd + 1.6 mM Put. Bar bars with different lowercase letters are significantly different by Duncan’s test (*p<* 0.05). Data are presented as the mean of five independent mean ± SD.

In Chang 6475, the SOD and POD activities were significantly increased by 18.06% and 22.23%, respectively (**Figure 7**); However, there was no significant difference in enzyme activities of Cd stress (T0) on Changmai 4013. Exogenous Put significantly enhanced the CAT activity of Changmai 4013, reaching its maximum at the T3 treatment, which represented a 34.50% increase compared to T0 (*P<* 0.05). In Chang 6475, the SOD and CAT activities peaked at T3, showing significant (*P<* 0.05) increases of 12.19% and 37.40%, respectively, while POD activity peaked at T2, showing a significant (*P<* 0.05) increase of 12.54% compared to T0. These findings suggest that Put seed soaking significantly mitigates oxidative stress in the sensitive variety Chang 6475 by enhancing antioxidant capacity.

**Figure 8:**
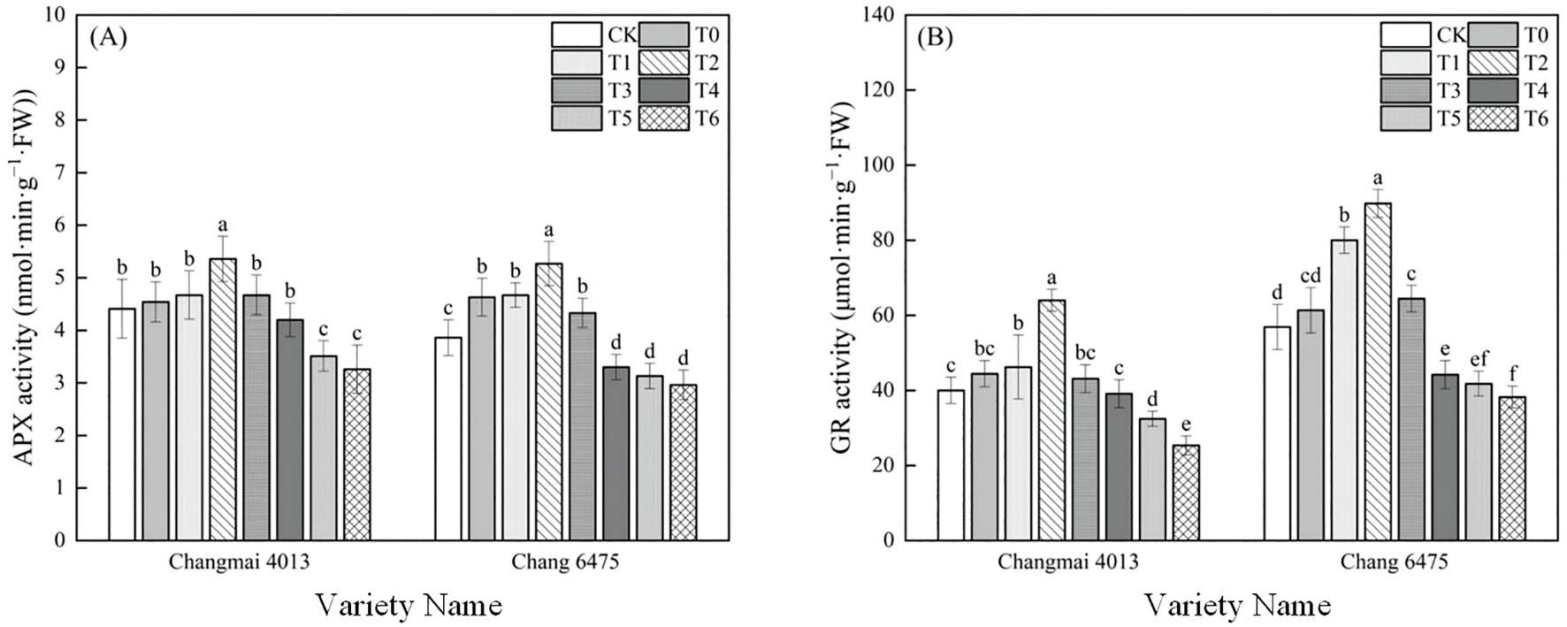
The impact of Put on **(A)** ascorbate peroxidase activity (APX), **(B)** glutathione reductase activity (GR) of Changmai 4013 and Chang 6475 under Cd stress. CK: Distilled water; T0: Cd + 0 mM Put; T1: Cd + 0.05 mM Put; T2: Cd + 0.1 mM Put; T3: Cd + 0.2 mM Put; T4: Cd + 0.4 mM Put; T5: Cd + 0.8 mM Put; T6: Cd + 1.6 mM Put. Bar bars with different lowercase letters are significantly different by Duncan’s test (*p<* 0.05). Data are presented as the mean of five independent mean ± SD.

The APX activity of Chang 6475 under Cd stress (T0) was significantly (*P<* 0.05) elevated by 19.95% compared to CK (**Figure 8**). Compared with T0 treatment, APX and GR activities of Changmai 4013 were significantly enhanced by 18.06% and 44.01% in T2 treatment; APX and GR activities of Chang 6475 were significantly (*P<* 0.05) enhanced by 13.82% and 46.39%. It was shown that Put has an essential action in regulating oxidative stress, which was particularly benefiting the sensitive variety Chang 6475.

**Figure 9:**
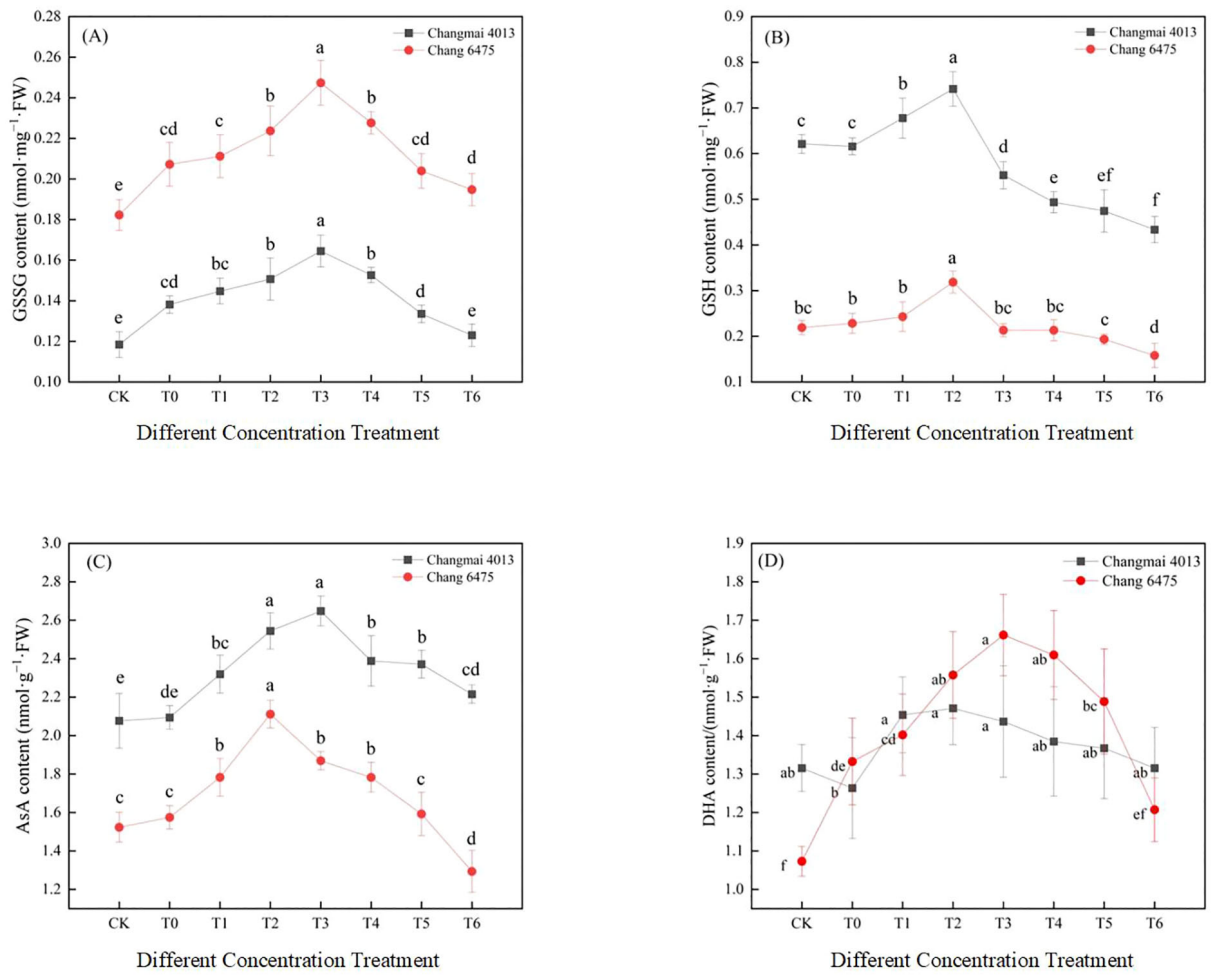
The impact of Put on **(A)** oxidized glutathione (GSSG), **(B)** reduced glutathione (GSH), **(C)** ascorbic acid (AsA), **(D)** dehydroascorbic acid (DHA) of Changmai 4013 and Chang 6475 under Cd stress. CK: Distilled water; T0: Cd + 0 mM Put; T1: Cd + 0.05 mM Put; T2: Cd + 0.1 mM Put; T3: Cd + 0.2 mM Put; T4: Cd + 0.4 mM Put; T5: Cd + 0.8 mM Put; T6: Cd + 1.6 mM Put. Bar bars with different lowercase letters are significantly different by Duncan’s test (*p<* 0.05). Data are presented as the mean of five independent mean ± SD.

Under Cd stress (T0), the levels of GSSG and AsA in Changmai 4013 increased significantly (*P<* 0.05) by 16.72% and 0.84%, respectively, compared to CK (**Figure 9**). In contrast, for Chang 6475, the GSSG and DHA contents increased significantly (*P<* 0.05) by 13.72% and 24.19%, respectively, compared to CK. Under different Put treatments, the GSSG and AsA contents in Changmai 4013 peaked at the T3 treatment, with significant (*P<* 0.05) increases of 19.03% and 26.44%, respectively, compared to T0. The GSH and DHA contents were highest at T2, with increases of 20.40% and 16.44%, respectively, compared to T0. For Chang 6475, the GSSG and DHA contents peaked at T3, with significant (*P<* 0.05) increases of 19.40% and 24.68%, respectively, compared to T0. Meanwhile, the GSH and AsA contents were maximized at T2, with significant (*P<* 0.05) increases of 39.47% and 34.07%, respectively. The promotion effect of Put treatment was more pronounced in Chang 6475 compared with Changmai 4013.

### 3.9 Spd, Spm and Put content

**Figure 10:**
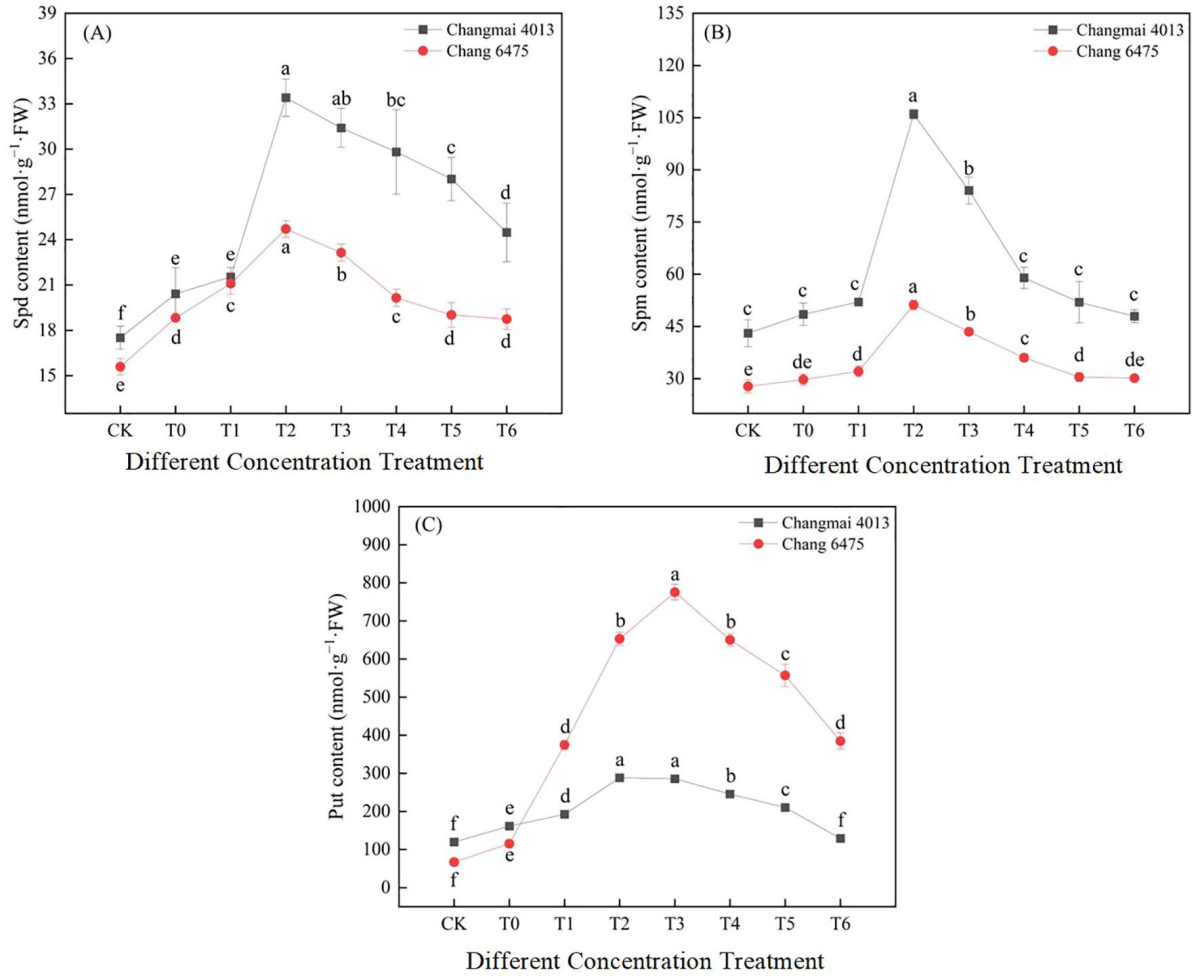
The impact of Put on **(A)** spermidine (Spd), **(B)** spermine (Spm), **(C)** putrescine (Put) of Changmai 4013 and Chang 6475 under Cd stress. CK: Distilled water; T0: Cd + 0 mM Put; T1: Cd + 0.05 mM Put; T2: Cd + 0.1 mM Put; T3: Cd + 0.2 mM Put; T4: Cd + 0.4 mM Put; T5: Cd + 0.8 mM Put; T6: Cd + 1.6 mM Put. Bar bars with different lowercase letters are significantly different by Duncan’s test (*p<* 0.05). Data are presented as the mean of three independent mean ± SD.

Under Cd stress (T0), Spd and Put content in both Changmai 4013 and Chang 6475 increased significantly (*P<* 0.05) compared to CK (**Figure 10**). Under different Put treatments, Spd, Spm, and Put content in Changmai 4013 peaked at T2, with significant (*P<* 0.05) increases of 63.58%, 118.63%, and 78.41%, respectively, compared to T0. For Chang 6475, Spd and Spm content peaked at T2, with significant (*P<* 0.05) increases of 31.26% and 72.31%, respectively, compared to T0, whereas Put content maximized at T3 with a remarkable 571.26% increase compared to T0. The stimulatory effect of exogenous Put on endogenous polyamine synthesis was more efficient in Chang 6475 than in Changmai 4013.

### 3.10 Evaluation of related traits under Put treatment

Under Cd stress, germination indices during the wheat germination period, along with seedling growth morphology, root characteristics, and physiological parameters under different concentrations of Put treatment, were used as the basis for a comprehensive evaluation of Changmai 4013 and Chang 6475 using the MV method (**Table 5**; **Supplementary Tables S3**, **S4**). Based on their membership values (MV), Changmai 4013 and Chang 6475 were ranked in the following order from highest to lowest: T2 > T3 > T1 > T4 > T0 > T5 > T6 > CK. Both varieties showed a consistent trend, with the T2 treatment yielding the highest MV. Therefore, a 0.1 mM Put concentration was determined to be the optimal concentration. Moreover, under CK and T0 treatments, the MV value of the Cd-sensitive variety Chang 6475 was lower than that of the Cd-tolerant variety Changmai 4013. However, under Put treatment, especially under T2 treatment, the MV value of the Cd-sensitive variety Chang 6475 was higher than that of the Cd-tolerant variety Changmai 4013, indicating that Put priming had a better mitigating effect on the Cd-sensitive variety.

**Table 5:** Evaluation of different Put concentrations using the membership function value method.

Variety	Treatment	MV	Rank
Changmai 4013	CK	0.317	8
	T0	0.512	5
	T1	0.552	3
	T2	0.758	1
	T3	0.668	2
	T4	0.552	4
	T5	0.474	6
	T6	0.354	7
Chang 6475	CK	0.293	8
	T0	0.501	5
	T1	0.589	3
	T2	0.761	1
	T3	0.719	2
	T4	0.581	4
	T5	0.479	6
	T6	0.414	7

### 3.11 Multivariate analysis of related traits under Put treatment

**Figure 11:**
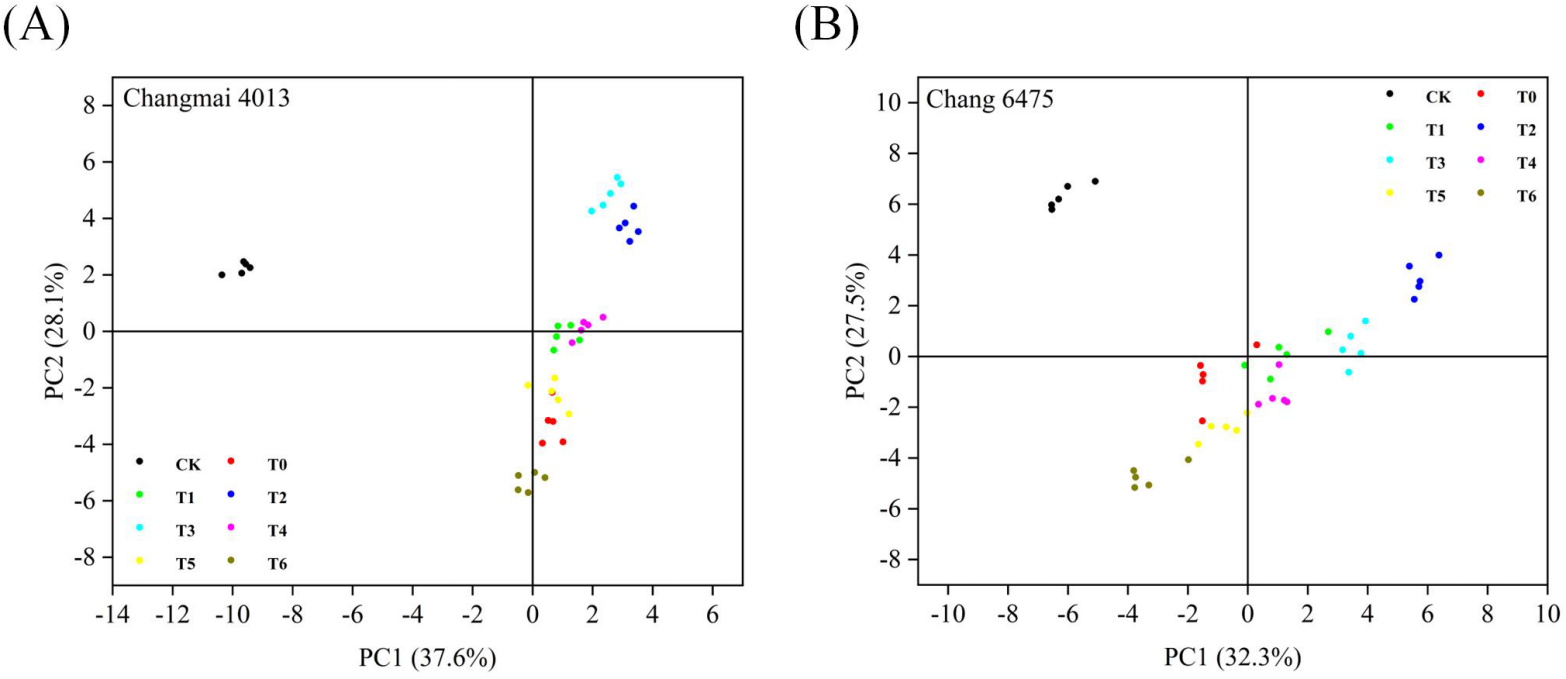
Principal component analysis of Changmai 4013 and Chang 6475 under Put treatment pairs. **(A)** Cd-tolerant variety Changmai 4013; **(B)** Cd-sensitive variety Chang 6475. CK: Distilled water; T0: Cd + 0 mM Put; T1: Cd + 0.05 mM Put; T2: Cd + 0.1 mM Put; T3: Cd + 0.2 mM Put; T4: Cd + 0.4 mM Put; T5: Cd + 0.8 mM Put; T6: Cd + 1.6 mM Put.

In this study, the physiological indices under different treatment conditions were compared by principal component analysis to reveal the pattern of variation and differences among treatment groups. **Figures 11A, B** show the distributions of the first and second principal components, respectively, explaining 37.6% and 28.1% of the variance in the data in Changmai 4013 and 32.3% and 27.5% of the variance in the data in Chang 6475, respectively. Furthermore, distinct separation was observed between the CK group and all treatment groups in both cultivars, indicating significant effects of Put treatment on wheat seedlings. Specifically, under Cd stress, data points for Changmai 4013 exhibited a gradual rightward shift (positive direction) along PC1 with increasing Put concentrations (T0-T6), suggesting partial mitigation of Cdinduced negative effects by Put application. In contrast, Chang 6475 demonstrated a more complex response pattern, while data points under medium-low Put concentrations (T1-T4) were evenly distributed, indicative of protective effects, those under high concentrations (T5, T6) shifted leftward (negative direction), implying potential exacerbation of Cd toxicity. Additionally, stronger similarity among Put treatment groups in Chang 6475 suggests greater commonality in its response to varying treatment conditions.

**Figure 12:**
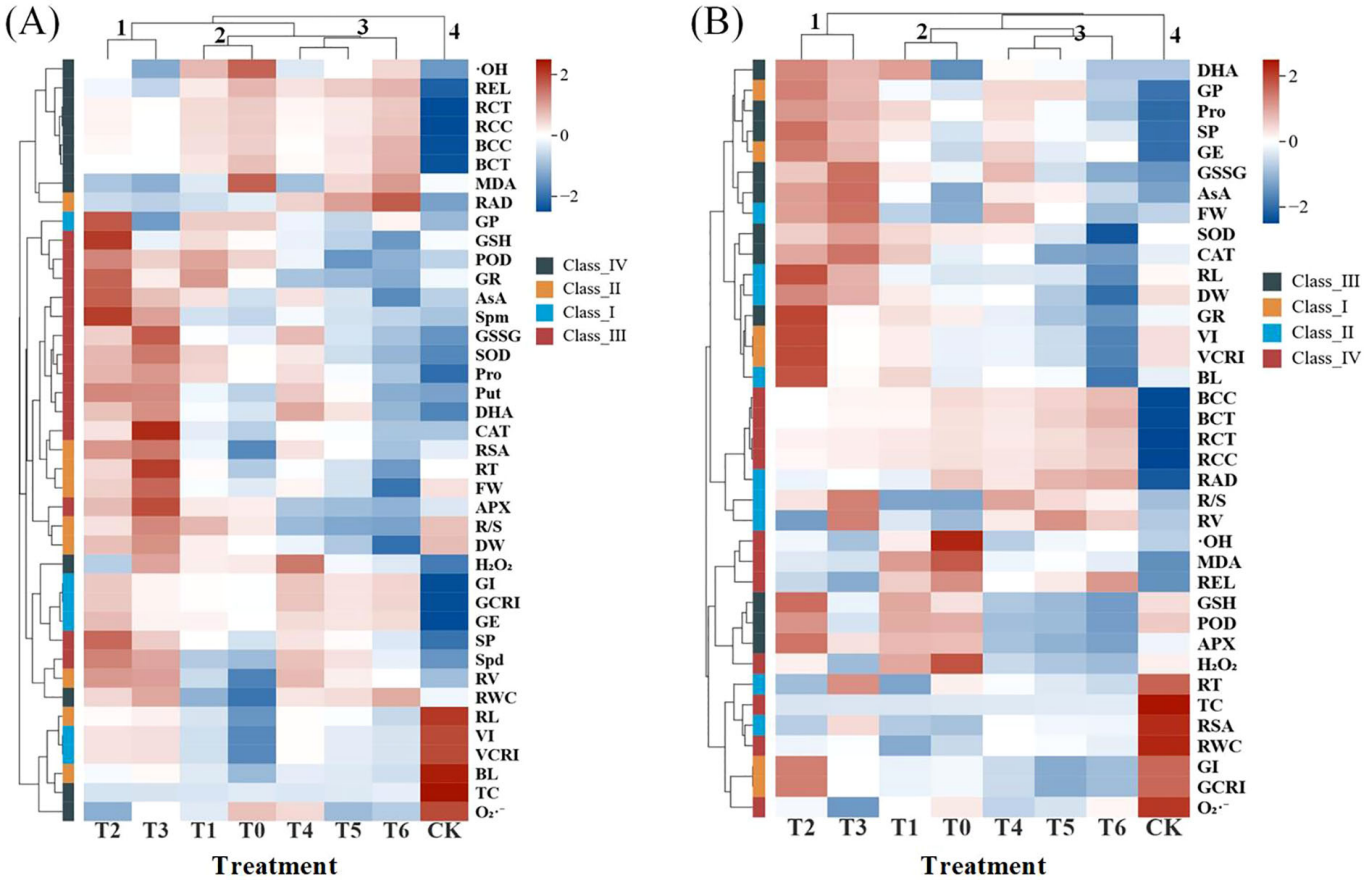
Hierarchical clustering analysis of Put treatment on Cd stress in Changmai 4013 and Chang 6475. Blue color indicates higher values and red color indicates lower values. **(A)** Cd-tolerant variety Changmai 4013; **(B)** Cd-sensitive variety Chang 6475. CK: Distilled water; T0: Cd + 0 mM Put; T1: Cd + 0.05 mM Put; T2: Cd + 0.1 mM Put; T3: Cd + 0.2 mM Put; T4: Cd + 0.4 mM Put; T5: Cd + 0.8 mM Put; T6: Cd + 1.6 mM Put. GE, germination energy; GP, germination percentage; GI, germination index; VI, vigor index; GCRI, germination Cd resistance index; VCRI, vigor Cd resistance index; RL, root length; BL, bud length; FW, fresh weight; DW, dry weight; R/S, root-to-shoot ratio; RWC, relative water content; REL, relative electrical conductivity; RSA, root surface area; RV, root volume; RT, number of root tips; RAD, root average diameter; BCC, bud Cd concentration; RCC, root Cd concentration; BCT, bud Cd content; RCT, root Cd content; TF, translocation factor; SOD, superoxide dismutase activity; POD, peroxidase activity; CAT, catalase activity; APX, ascorbate peroxidase activity; GR, glutathione reductase activity; SP, soluble protein; O_2_·^–^, superoxide anion rate; MDA, malondialdehyde; H_2_O_2_, hydrogen peroxide; Pro, free proline; ·OH, hydroxyl radical; GSSG, oxidized glutathione; GSH, reduced glutathione; AsA, ascorbic acid; DHA, dehydroascorbic acid; Spd, spermidine; Spm, spermine; Put, putrescine.

Subsequently, hierarchical cluster analysis was performed on the physiological indices of wheat varieties Changmai 4013 and Chang 6475, respectively (**Figure 12**). In the hierarchical clustering, both varieties grouped the different treatments into four clusters: Cluster 1 (T2 and T3), Cluster 2 (T1 and T0), Cluster 3 (T4, T5, and T6), and Cluster 4 (CK). Cd stress (T0 treatment) resulted in higher values of REL, ·OH, H_2_O_2_, Cd content, and MDA in both varieties. Exogenous Put treatment reduced these values while increasing the values of germination parameters, growth parameters, antioxidant enzyme activities, antioxidants, and endogenous PAs (Spd, Spm, Put) in both cultivars. The T2 and T3 treatments collectively demonstrated the most significant advantages, indicating these treatments promoted plant growth, germination, and Cd resistance. Furthermore, the values of all physiological indices under T4-T6 treatments were significantly lower compared to other treatments, suggesting that excessively high Put concentrations have an inhibitory effect on Cd-sensitive varieties.

**Figure 13:**
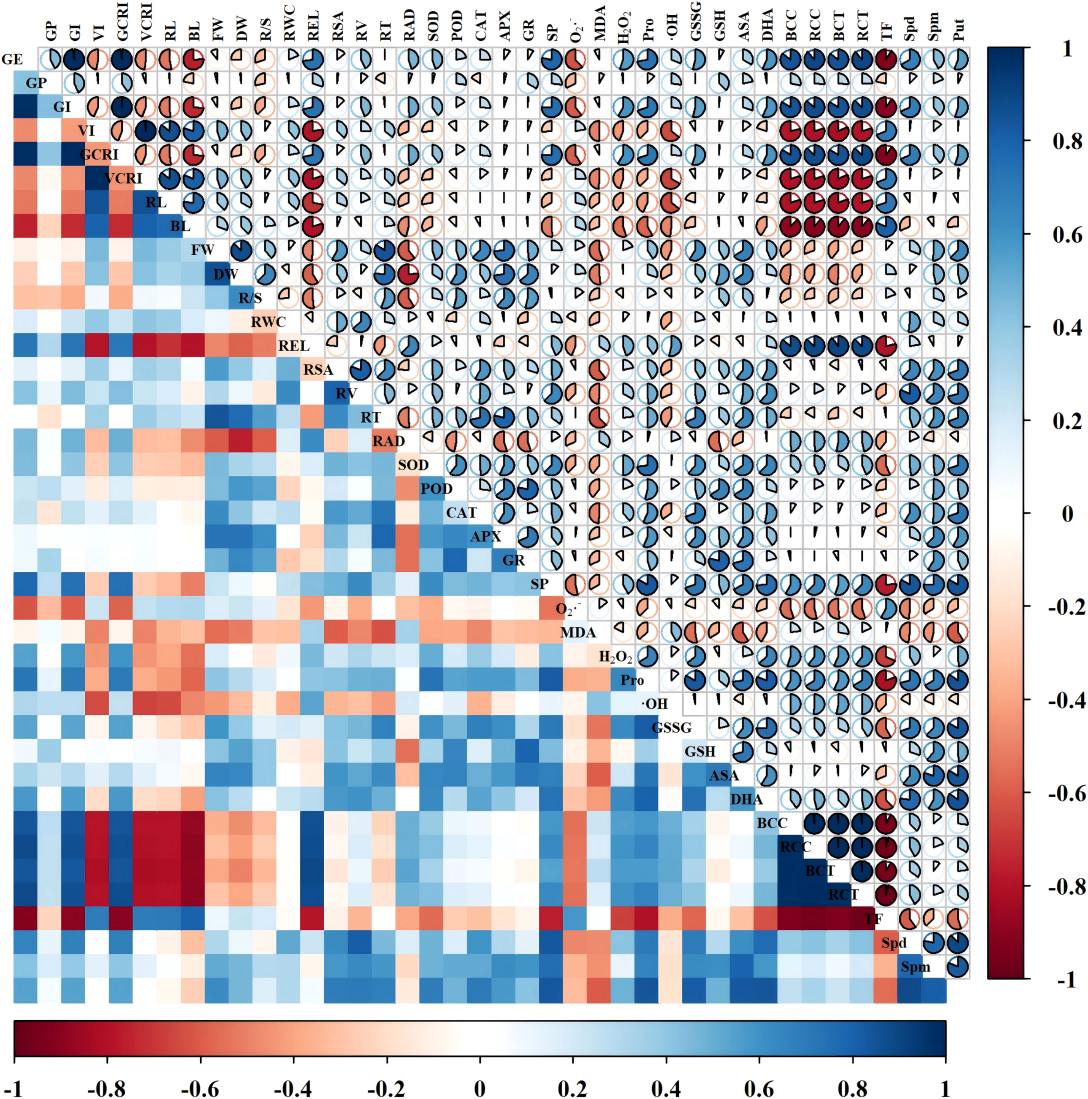
Correlation analysis of Put treatments on Changmai 4013 under Cd stress. Blue color indicates positive correlation, red color indicates negative correlation, and sector area indicates the magnitude of the correlation coefficient. GE, germination energy; GP, germination percentage; GI, germination index; VI, vigor index; GCRI, germination Cd resistance index; VCRI, vigor Cd resistance index; RL, root length; BL, bud length; FW, fresh weight; DW, dry weight; R/S, root-to-shoot ratio; RWC, relative water content; REL, relative electrical conductivity; RSA, root surface area; RV, root volume; RT, number of root tips; RAD, root average diameter; BCC, bud Cd concentration; RCC, root Cd concentration; BCT, bud Cd content; RCT, root Cd content; TF, translocation factor; SOD, superoxide dismutase activity; POD, peroxidase activity; CAT, catalase activity; APX, ascorbate peroxidase activity; GR, glutathione reductase activity; SP, soluble protein; O_2_·^–^, superoxide anion rate; MDA, malondialdehyde; H_2_O_2_, hydrogen peroxide; Pro, free proline; ·OH, hydroxyl radical; GSSG, oxidized glutathione; GSH, reduced glutathione; AsA, ascorbic acid; DHA, dehydroascorbic acid; Spd, spermidine; Spm, spermine; Put, putrescine.

**Figure 14:**
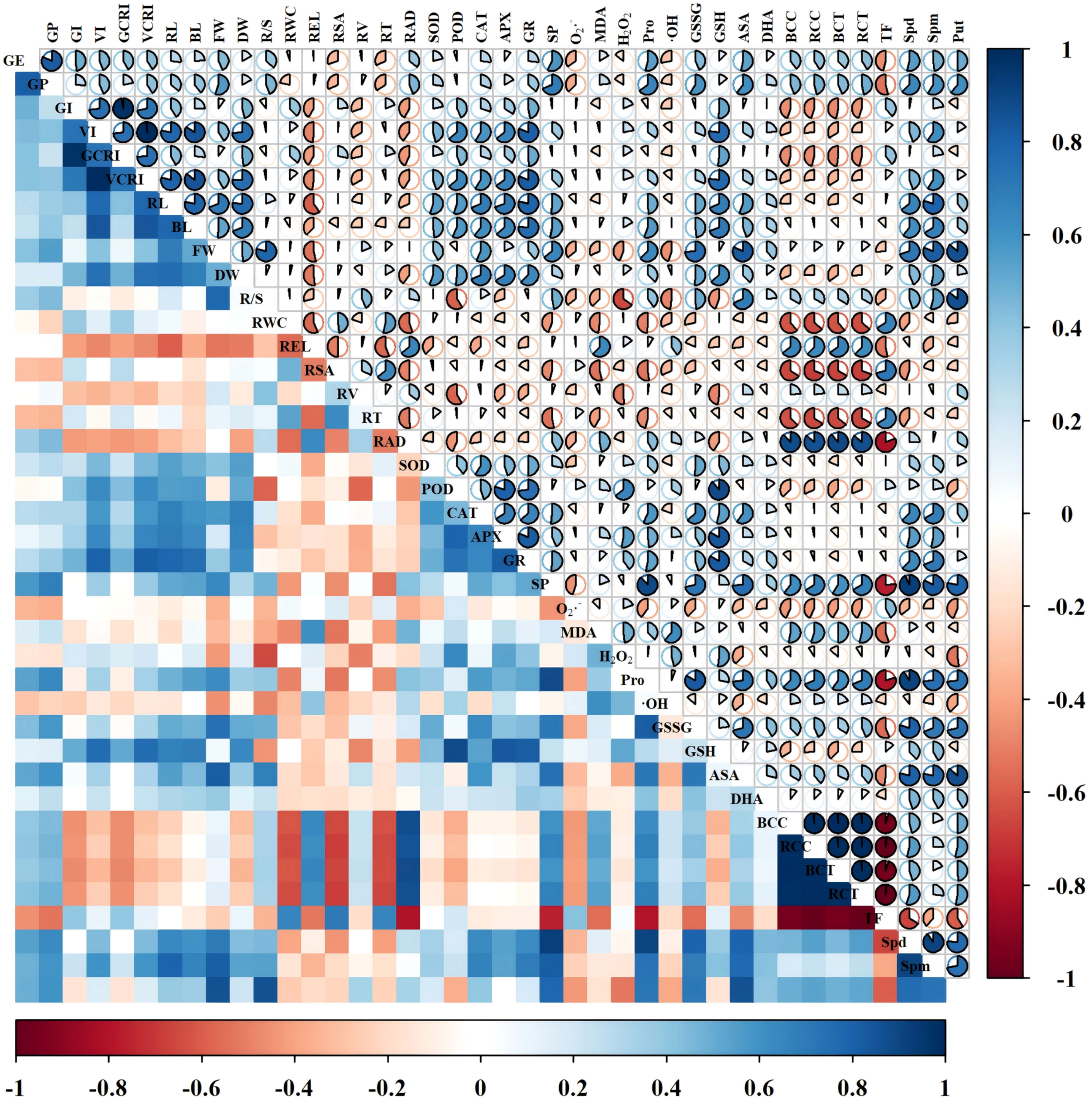
The impact of Put on **(A)** root Cd concentration (RCC), **(B)** bud Cd concentration (BCC), **(C)** root Cd content (RCT), **(D)** bud Cd content (BCT), **(E)** translocation factor (TF) of Changmai 4013 and Chang 6475 under Cd stress. CK: Distilled water; T0: Cd + 0 mM Put; T1: Cd + 0.05 mM Put; T2: Cd + 0.1 mM Put; T3: Cd + 0.2 mM Put; T4: Cd + 0.4 mM Put; T5: Cd + 0.8 mM Put; T6: Cd + 1.6 mM Put. Bar bars with different lowercase letters are significantly different by Duncan’'s test (*p<* 0.05). Data are presented as the mean of five independent mean ± SD.

The physiological indicators were correlated by constructing a correlation matrix (**Figures 13**, **14**). We can see that in the Changmai 4013, fresh weight and dry weight showed a significant positive correlation with antioxidant enzyme activities (SOD, POD, CAT, APX, and GR) and antioxidant substances (GSSG, GSH, AsA, and DHA), and endogenous PAs content (Spm, Put). Growth indicators (VI, VCRI, RL, BL, FW, and DW) were significantly negatively correlated with oxidative stress markers (REL, MDA, H_2_O_2_, Pro, and ·OH) and Cd accumulation parameters (BCC, RCC, BCT, RCT). For the Chang 6475, growth indicators showed a significant positive correlation with antioxidant enzyme activities, antioxidant substances, and endogenous PAs content (Spd, Spm, Put), while they were significantly negatively correlated with REL and root conditions. The root parameters of Chang 6475 were more severely affected by stress. Additionally, relative water content (RWC), root surface area (RSA), and root tip number (RT) showed significant negative correlations with Cd accumulation parameters (BCC, RCC, BCT, RCT). Compared to Chang 6475, the Cd-tolerant variety Changmai 4013 exhibited more significant negative correlations (red fanshaped areas), especially in germination and growth indicators. This suggests that Changmai 4013 can better maintain its normal physiological and biochemical functions to resist cadmium-induced oxidative damage. In contrast, the Cd-sensitive variety Chang 6475 showed more significant positive correlations (blue fan-shaped areas), particularly in the antioxidant system. This indicates that Chang 6475 relies on stronger antioxidant enzyme activity and the accumulation of non-enzymatic antioxidants to cope with cadmium-induced oxidative stress, thereby mitigating its negative effects on growth and development to some extent.

## 4 Discussion

Germination is the stage in the entire reproductive period of plants that is most sensitive to the perception of the external environment (Lu et al., 2021). During this stage, Cd stress induces damage to cell membranes in seedlings, causing alterations in the composition of the internal protective enzyme system and changes in its activity, which in turn affects physiological processes (Du et al., 2024). This study shows that Cd stress promotes seed germination but significantly inhibits the growth of the radicle and hypocotyl. This could be attributed to the stimulatory effect of Cd on plants, which might accelerate plant growth by inducing the synthesis and accumulation of phytohormones. However, Cd stress also imposes physiological limitations on the plant, reducing seedling growth performance (Zaari Jabri et al., 2024). Moreover, exogenous Put treatment significantly enhanced seed vigor and Cd tolerance, promoting germination rate, growth, and the overall health of wheat seedlings. The treatment with 0.1-0.2 mmol · L^−1^ Put showed the most significant effect, which aligns with the findings of Ebeed et al. (2017) that 0.1 mmol · L^−1^ polyamine treatment positively influenced dry weight, fresh weight, and redox status in wheat under stress conditions. This likely occurs because Put enhances enzyme activity in the radicle and hypocotyl, or increases cellulose content, thus mitigating Cd-induced damage to cell membranes (Tajti et al., 2018). Additionally, this study revealed that exogenous Put seed soaking was more effective in improving root morphology of the Cd-sensitive variety Chang 6475 compared to the tolerant variety. It enhanced shoot length, root surface area, and biomass of wheat seedlings, improved growth status, activated lateral root primordium development signaling, and significantly increased lateral root density. This indicates that exogenous Put can more effectively alleviate the inhibitory effects on seedling growth in stress-sensitive cultivars, while highlighting its divergent regulatory mechanisms between stress-tolerant and stress-sensitive varieties under stress conditions (Guo et al., 2018). The precise action mechanisms of Put in mitigating Cd toxicity in plant root systems remain to be elucidated.

After heavy metal Cd enters plants, it tends to accumulate more easily in the roots, especially in crops. In this study, the Cd concentration in the roots of wheat seedlings was significantly higher than that in the shoots (**Figure 14**), which is a common phenomenon (Murtaza et al., 2019). Meanwhile, in this study, the application of exogenous Put effectively reduced Cd absorption in the roots and Cd concentration in the shoots. The effect of this interaction on reducing shoot Cd concentration was more pronounced, indicating that Put can inhibit the translocation of heavy metal Cd from roots to shoots. Additionally, the much higher Cd content in roots compared to shoots may also be attributed to Put promoting the accelerated transport of heavy metals in root cells through phytochelatins (PCs), thereby alleviating their toxicity. As reported by Di et al. (2023), when heavy metal Cd enters plants, it is chelated by reduced glutathione (GSH) in the roots to form a metal-PC complex, which is then transported into vacuoles. This process stores Cd in root cells, alleviating its toxicity and reducing damage to other parts of the plant caused by long-distance transport of heavy metals. The specific cellular mechanisms involved in this study still require further investigation.

Under the adversity environment, plant cells need to rapidly initiate the biosynthesis of osmotic regulators such as Pro and other organic osmolytes, which effectively regulate osmotic pressure and maintain water balance (Zhou et al., 2016). This phenomenon suggests that under Cd toxicity, exogenous Put may synergistically enhance the biosynthesis of osmotic regulators, such as SP and Pro, thereby maintaining cellular osmotic balance and stabilizing macromolecules like proteins to resist stress-induced damage, which is basically in line with the results of the research by Gu et al (Gu et al., 2022). MDA content, relative water content, and relative conductivity are often used as key indicators to characterize the permeability and integrity of cell membranes, which can reflect the degree of cell membrane damage (Fan et al., 2015). In this research, Cd stress resulted in a decrease in relative water and a significant increase in relative conductivity and MDA content in wheat seedlings, likely due to the Cd-induced generation of free radicals that lead to lipid peroxidation and membrane damage (Heile et al., 2021). Application of exogenous Put under Cd stress significantly reduced relative conductivity and MDA content while increasing relative water content, indicating that Put helps retain water in plant tissues and maintains cell membrane stability under stress. Similarly, under drought stress conditions in tomatoes, the application of exogenous substances similarly demonstrated significant reductions in leaf MDA content alongside increased relative water content (Wang et al., 2022), a pattern that closely aligns with the observed decreases in MDA levels and enhanced water status in cadmium-stressed rice plants in the current study. Thus, it is hypothesized that Put participates in the osmotic regulation pathway under Cd stress to preserve membrane integrity and improve water status in plants.

The crucial mechanism by which polyamines regulate plant resistance to stress lies in their dual action of activating the antioxidant enzyme system and repairing cell membrane integrity (Islam et al., 2020). In this research, Cd caused a surge in H_2_O_2_, ·OH, and O_2_·^–^ production rates in wheat seedlings, indicating that ROS metabolic imbalance is a key factor in Cd-induced toxicity affecting wheat growth and development (Ma et al., 2024). Plants can effectively maintain redox homeostasis through their protective enzyme systems, thereby resisting the oxidative damage triggered by Cd stress (Feng et al., 2023). Among antioxidant enzymes, SOD converts O_2_·^–^ produced under stress into H_2_O_2_ and O_2_ (Jiang et al., 2019). Subsequently, POD reduces H_2_O_2_ to H_2_O and O_2_, effectively mitigating the damage caused by free radicals (Zhang et al., 2015). CAT works synergistically with POD to further participate in ROS metabolism (Yan et al., 2010). Therefore, the ability of protective enzymes such as SOD and POD to maintain high activities is important for plants under adverse environments. In this research, all antioxidant enzyme activities were significantly increased by exogenous Put treatment, alleviated the accumulation of H_2_O_2_, O_2_·^–^, and ·OH, and reduced oxidative damage caused by Cd stress, thus supporting normal wheat growth (Yang et al., 2024). Previous studies have demonstrated that under cadmium-lead combined stress, the application of spermidine (Spd) to rice seedlings yielded similar results, manifested as increased activity of ROSscavenging enzymes, reduced ROS, and enhanced growth vigor in rice seedlings (Gu et al., 2022). These findings suggest that the application of polyamines promotes crop growth by activating the antioxidant system to counteract oxidative stress induced by heavy metal stress, thereby mitigating the effects of heavy metal contamination on crop seedlings.

The realization of physiological functions in the AsA-GSH cycle system primarily depends on the critical catalytic roles of APX and GR (Kar, 2011). Through synergistic regulation of AsA regeneration and GSH redox cycling, these enzymes collectively ensure the efficient operation of the antioxidant defense system (Akram et al., 2017). Under stress conditions, exogenous Put can activate the APX/GR enzyme cascade, significantly enhancing the AsA-GSH cycle flux, efficiently scavenging stress-induced ROS, blocking lipid peroxidation chain reactions, and preserving cell membrane integrity (Chowardhara et al., 2022). In this research, Cd stress increased the activity of APX and GR and the contents of AsA and GSSG in wheat. Put treatment further increased the activity and content of these metabolic enzymes, indicating that an appropriate concentration of Put can effectively strengthen the metabolic function of this cyclic loop, enhancing its redox regulatory capacity of this system and, thus, improving the physiological mechanisms underlying wheat’s Cd tolerance. Moreover, the effect of Put was more significant on the sensitive variety Chang 6475, likely due to its heightened sensitivity to environmental conditions, making exogenous regulators more effective in mitigating oxidative stress under environmental stress (Guo et al., 2018).

PAs serve as crucial protective molecules in plants under stress, playing vital roles in stabilizing cellular structures and enhancing stress tolerance (Kaur and Das, 2023). Our results demonstrate that Cd stress (T0) significantly induced the accumulation of endogenous Spd and Put in both wheat varieties, Changmai 4013 and Chang 6475. This aligns with the established role of PAs in mitigating heavy metal toxicity through membrane integrity maintenance and ROS scavenging (Biondi et al., 2022). Notably, seed soaking with exogenous Put (T1-T6) under Cd stress significantly elevated endogenous Spd, Spm, and Put contents in both cultivars, reaching peak levels at T2-T3 treatments. Chang 6475 exhibited a significantly higher increase in endogenous Put content than Changmai 4013 at T3 treatment, indicating that this sensitive variety more efficiently activates endogenous Put biosynthesis pathways. This observation closely aligns with mechanisms reported for exogenous substance regulation of PA metabolism in maize under Cd stress (Piao et al., 2022), suggesting potential differentiation in the activity or expression of key PA metabolic enzymes (ADC/ODC) between varieties. Similarly, exogenous Spd treatment in rice under Al stress significantly elevated endogenous PAs and alleviated oxidative damage (Jiang et al., 2021), demonstrating a PA-mediated stress protection mechanism highly consistent with the PA accumulation patterns observed in our study under Cd stress. Therefore, we propose that exogenous Put may provide multi-layered protection against Cd stress by variety-specifically activating PA biosynthesis pathways, thereby synergistically enhancing membrane stability, osmotic/ionic homeostasis, and antioxidant defense. Future research should focus on elucidating the key molecular mechanisms by which exogenous Put regulates polyamine biosynthesis and antioxidant defense, and explore its application potential in developing resistance and agronomic management strategies for crops in Cd-contaminated soils.

**Figure 15:**
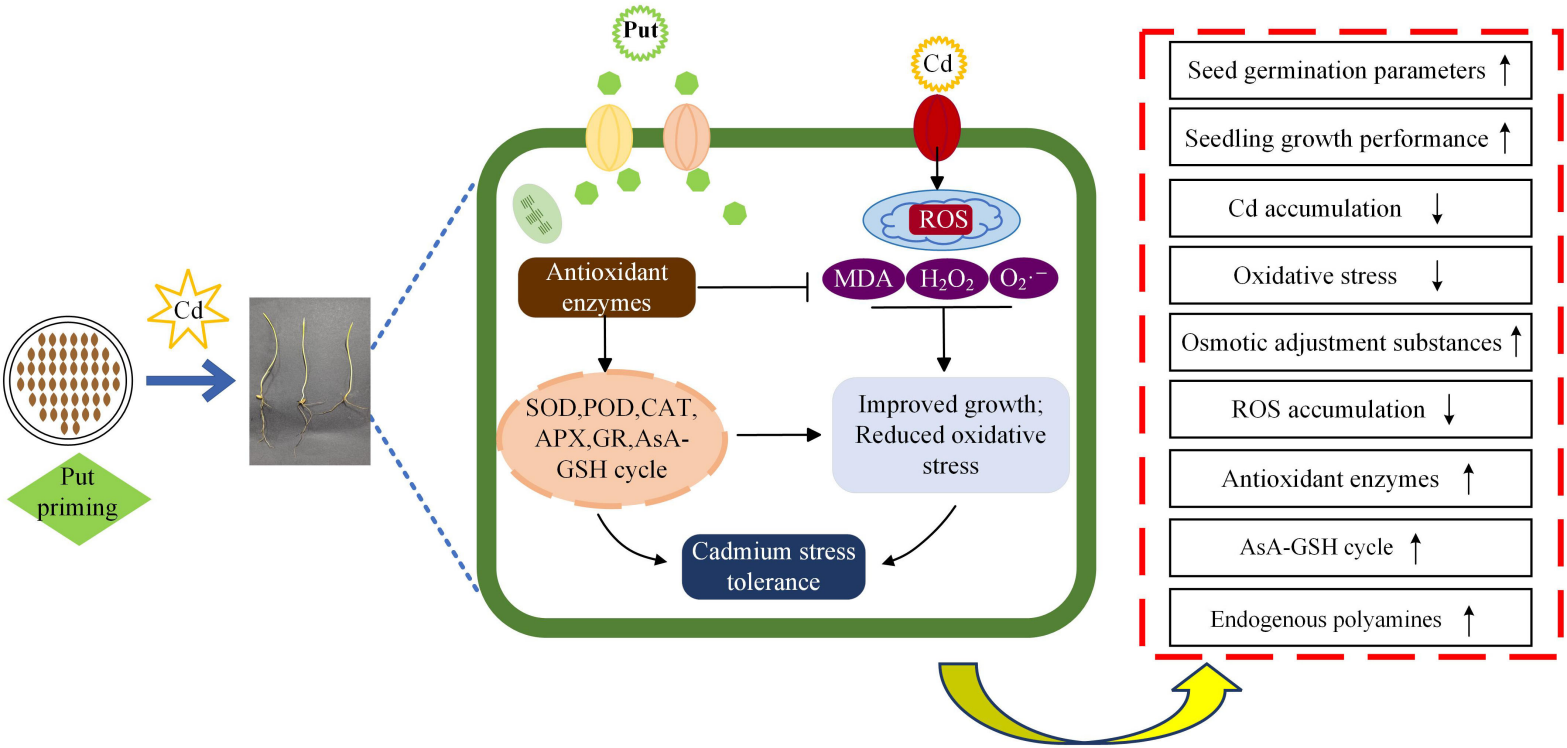
Schematic modeling of Put-induced mitigation of Cd stress effects in wheat seedlings.

Principal component, hierarchical cluster, and correlation heatmap analysis were performed for each parameter. Exogenous Put reduced the toxicity produced by Cd in wheat. Wheat seedlings under Cd stress showed lower values of growth parameters and higher values of MDA, H_2_O_2_, O_2_·^–^, and ·OH contents, whereas seedlings under Put treatment showed the opposite trend, suggesting that Put effectively scavenges lipid peroxides generated by Cd stress in wheat seedlings, while enhancing the plant’s protective capacity. Specifically, exogenous Put promotes the plant’s endogenous PA biosynthesis pathway, thereby activating the antioxidant defense system, rapidly decomposing accumulated lipid peroxides, and enhancing cell membrane damage repair capacity. This process not only helps plants resist heavy metal-induced damage more effectively but also promotes the recovery of damaged tissues, enabling seedlings to maintain normal growth under stress (**Figure 15**). Additionally, this study used multivariate analysis to compare the responses of Cd-sensitive (Chang 6475) and Cd-tolerant (Changmai 4013) wheat varieties to exogenous Put. Principal component analysis (PCA) revealed that Put-treated groups of the sensitive variety formed tightly clustered distributions, suggesting concentration-dependent responses. This pattern may reflect their reliance on exogenous Put to compensate for weaker endogenous antioxidant systems. Hierarchical clustering further indicated that low concentrations of Put (0.1–0.2 mM) significantly enhanced Cd resistance in the sensitive variety, marked by coordinated increases in endogenous polyamine content, antioxidant markers (SOD, CAT, GSH), and growth parameters. However, higher concentrations (T4–T6) likely reduced effectiveness due to cellular overstress or signaling disruption. Correlation matrix analysis revealed significant positive correlations (blue sectors) between endogenous polyamine content (Spd, Spm, Put), antioxidant enzymes (CAT, APX), non-enzymatic antioxidants (GSH, AsA), and growth metrics in the sensitive variety. This demonstrates that the variety mitigates Cd toxicity by prioritizing antioxidant system activation over merely maintaining physiological homeostasis, with stress resistance efficacy particularly dependent on Put regulation. In contrast, the tolerant variety relied on intrinsic resistance mechanisms (cell membrane stability), showing stronger negative correlations (red sectors) between physiological stability markers (REL) and growth. These findings support targeted Put application in Cd-sensitive crops.

Simultaneously, this study employed the Membership Function (MV) method to comprehensively evaluate the effect of exogenous Put on alleviating Cd stress. This method standardizes germination parameters, root traits, and physiological-biochemical indices to eliminate measurement scale differences and integrate complex biological responses, enabling objective quantification of plant stress resistance (Shen et al., 2024). Results demonstrated that 0.1 mM Put significantly increased MV values in both Changmai 4013 and Chang 6475 cultivars, indicating its optimal concentration for Cd toxicity mitigation. Notably, the Cd-sensitive cultivar Chang 6475 exhibited lower MV values than the Cd-tolerant Changmai 4013 under CK and Cd stress (T0) conditions. However, its MV value surpassed the tolerant cultivar under T2 treatment, suggesting enhanced physiological restoration effects of Put seed-soaking in sensitive genotypes. This phenomenon may be attributed to exogenous Put effectively elevating the levels of protective endogenous PAs (Spd, Spm, Put) under Cd stress. This elevation potentially activates the antioxidant defense system or enhances heavy metal chelation pathways, thereby efficiently compensating for stress tolerance deficiencies and achieving synergistic improvements in morphological and physiological traits, consistent with findings by Zhao et al. (2024). Future studies should integrate analyses of key gene expression in PA metabolism pathways and Cd subcellular distribution mapping to further elucidate the details of Put concentration-dependent regulatory networks and their genotype-specific mechanisms.

## 5 Conclusion

Under Cd stress, seed soaking with appropriate concentrations of Put effectively alleviated growth inhibition and oxidative damage in both wheat cultivars, though significant inter-varietal response differences were observed. We recommend prioritizing low-concentration Put (0.1-0.2 mM) to specifically enhance antioxidant defense systems in the Cd-sensitive cultivar (Chang 6475), while for the Cd-tolerant cultivar (Changmai 4013), emphasis should be placed on coordinating constitutive resistance with field adaptability rather than applying high-concentration interventions. Comprehensive evaluation using membership function analysis identified 0.1 mM Put as the optimal concentration for both cultivars. However, the distinct Cd-resistance mechanisms between cultivars require further molecular-level investigation to elucidate their differential regulatory pathways. This study provides cultivar-specific management strategies for mitigating Cd toxicity through polyamine regulation in wheat production systems.

## Data Availability

The original contributions presented in the study are included in the article/**Supplementary Material**. Further inquiries can be directed to the corresponding authors.
