# Supplementary material for: Exogenous putrescine modulates variety-specific cadmium tolerance in wheat seedlings: synergistic roles of antioxidant defense and physiological homeostasis
Source: Front Plant Sci. 2025 Sep 8;16:1600603. doi: 10.3389/fpls.2025.1600603 (PMC12452105; doi:10.3389/fpls.2025.1600603)
Supplement: Supplementary file 1 [file DataSheet1.pdf]

Table S1 Methods for Physiological Parameter Analysis

Index	Methodological procedure
Superoxide anion production rate ($O_2{\cdot}^-$)	A 0.1 g leaf tissue sample was soaked in 65 mM phosphate buffer (pH 7.8) and ground thoroughly. After centrifugation at 12,000×g for 15 min, the supernatant was mixed with 65 mM phosphate buffer (pH 7.8) and 10 mM hydrochloric acid hydroxylamine and incubated at 25°C for 20 min. Then, 17 mM 4−aminobenzenesulfonic acid and 7 mM α−naphthylamine were added and mixed thoroughly. After incubating the mixture at 30 °C for 30 min, the absorbance was measured at 530 nm using a spectrophotometer.
Determination of Cd content	Approximately 0.1-0.4 g of dried root and shoot tissue samples were weighed and transferred into polytetrafluoroethylene (PTFE) digestion vessels, followed by the addition of 5 mL nitric acid for overnight soaking. After securing the inner lids and tightening the stainless steel outer casings, the vessels were placed in a constant-temperature drying oven and subjected to a graded heating program: maintained at 80°C for 2 h, increased to 120°C for another 2 h, then further elevated to 160°C for 4 h. The vessels were allowed to cool naturally to room temperature within the oven before opening. The digested solutions were subsequently heated to near-dryness for acid removal, then quantitatively transferred to 25 mL volumetric flasks. Residual material in the digestion vessels and lids was rinsed three times with small volumes of 1% nitric acid solution, with all washings combined into the volumetric flasks. The solutions were finally diluted to the mark with 1% nitric acid and homogenized for analysis. Reagent blank tests were conducted in parallel throughout the procedure. Cd concentrations were determined using inductively coupled plasma mass spectrometry (ICP-MS) in accordance with the Chinese national standard GB 5009.268-2016.
Evans blue staining	The tissue staining was performed using Evans blue staining method. The shoots of wheat seedlings were washed with ultrapure water and transferred into 15 mL centrifuge tubes containing 0.1% Evans blue staining solution. After vacuum infiltration until the shoots were completely submerged, the samples were kept in darkness for 5 h. Subsequently, the shoots were removed and rinsed with ultrapure water to eliminate adsorbed dye from the leaf surfaces. The samples were then placed in new 15 mL centrifuge tubes containing 95% ethanol and maintained for 24 h until complete destaining was achieved, followed by photographic documentation.

Hydrogen peroxide content (H_2O_2)	A 0.1 g leaf tissue sample was soaked in a mortar containing a 0.1% (w/v) trichloroacetic acid solution. After centrifugation at 12,000×*g* for 12 min, the supernatant was mixed with 10 mM potassium phosphate buffer (pH 7.0) and KI solution, and the absorbance was measured at 390 nm using a spectrophotometer.
Hydroxyl radical content (·OH)	A 0.1 g fresh sample was ground in 10 mM sodium phosphate buffer (pH 7.4) containing 15 mM 2-deoxy-D-ribose) to form a homogenate. After centrifugation at 10,000×*g* for 20 min, the supernatant was incubated at 37 °C for 2 h, mixed with 0.5% thiobarbituric acid and glacial acetic acid, and heated in a boiling water bath for 30 min. After cooling to 41 °C, the absorbance of the supernatant was measured at 550 nm.
Malondialdehyde content (MDA)	A 0.2 g fresh sample was mixed with a small amount of quartz sand and 0.1% trichloroacetic acid and then ground in an ice bath. Thereafter, 0.5% thiobarbituric acid was added, heated in a boiling water bath for 15 min, cooled to room temperature, and centrifuged at 3,000×*g* for 15 min. The absorbance of the supernatant was measured separately at 532 and 600 nm.
Soluble protein content (SP)	Fresh samples (0.2 g) were added to a pre-cooled 50 mM potassium phosphate buffer (pH 7.8) and ground into a homogenate in a pre-cooled mortar. The mixture was centrifuged at 12,000×*g* for 15 min at 4 °C, and the supernatant was mixed thoroughly with G−250 solution and allowed to stand for 2 min. The absorbance was measured at 595 nm using a spectrophotometer.
Free proline content (Pro)	A 0.2 g fresh sample was thoroughly ground on ice using a small amount of quartz sand and 3% sulfosalicylic acid. The homogenate was placed in a boiling water bath for 10 min, cooled, and centrifuged at 4,000×*g* for 10 min. The supernatant was mixed with glacial acetic acid and acid ninhydrin and heated in a boiling water bath for 30 min. After cooling to room temperature, toluene was added for extraction in the dark. The absorbance of the toluene phase (red) was measured at 520 nm using a spectrophotometer.

Antioxidant enzyme activity	A 0.2 g fresh sample was mixed with a small amount of quartz sand and 50 mM phosphate buffer (pH 7.8) and ground on ice. The mixture was centrifuged at 12,000×*g* and 4°C for 15 min. The supernatants were used to measure the activities of superoxide dismutase activity (SOD), peroxidase activity (POD), catalase activity (CAT), glutathione reductase activity (GR), and ascorbate peroxidase activity (APX).
AsA−GSH cycle product and substrate content	A 0.1 g fresh sample was ground into a homogenate using 5% sulfosalicylic acid in a pre-cooled mortar. The mixture was centrifuged at 10,000×*g* and 4°C for 15 min, and the supernatant was used to measure the contents of ascorbic acid (AsA), dehydroascorbic acid (DHA), reduced glutathione (GSH), and oxidized glutathione (GSSG).

Table S2 Ion pair information

Sample ID	Compound Name	Detection Mode	Retention Time (MIN)	Precursor Ion (M/Z)	Product Ion (M/Z)	DP(VOLTS)	Collision Energy(V)	Notes
R268_1	L-PHENYLALANINE	Positive	0.9	171.1	125.1	10	20	Quantifier Ion（Internal Standard）
R268_2	L-PHENYLALANINE	Positive	0.9	171.1	106	30	40	Qualifier Ion（Internal Standard）
Spermidine_1	Spermidine	Positive	0.4	146.2	72.1	30	25	Quantifier Ion
Spermidine_2	Spermidine	Positive	0.4	146.2	30	30	40	Qualifier Ion
Spermine_1	Spermine	Positive	0.4	203.2	129.2	20	15	Quantifier Ion
Spermine_2	Spermine	Positive	0.4	203.2	112.2	20	25	Qualifier Ion
Putrescine_1	Putrescine	Positive	0.4	89.1	72.1	20	15	Quantifier Ion
Putrescine_2	Putrescine	Positive	0.4	89.1	30.3	20	30	Qualifier Ion

Table S3　Comprehensive analysis of the effects of different concentrations of exogenous Put on Changmai 4013 under Cd stress

Index	CK	T0	T1	T2	T3	T4	T5	T6
GE	0.000	0.417	0.583	1.000	0.833	0.750	0.500	0.583
GP	0.000	0.455	0.545	1.000	0.818	0.727	0.727	0.364
GI	1.000	0.361	0.333	0.917	0.403	0.222	0.000	0.083
VI	0.579	0.402	0.533	1.000	0.486	0.410	0.322	0.000
GCRI	0.000	0.765	0.809	0.986	0.845	1.000	0.895	0.946
VCRI	1.000	0.000	0.327	0.561	0.572	0.482	0.379	0.346
RL	0.495	0.344	0.404	1.000	0.710	0.353	0.341	0.000
BL	0.428	0.425	0.634	1.000	0.534	0.504	0.475	0.000
FW	0.194	0.000	0.181	0.826	1.000	0.733	0.434	0.056
DW	0.548	0.437	0.591	1.000	0.689	0.563	0.314	0.000
R/S	0.134	0.000	0.009	0.613	1.000	0.858	0.646	0.554
RWC	1.000	0.180	0.000	0.283	0.320	0.341	0.314	0.265
REL	0.000	0.996	0.819	0.668	0.520	0.843	0.926	1.000
RSA	1.000	0.000	0.031	0.052	0.418	0.264	0.220	0.216
RV	0.206	0.138	0.356	0.000	1.000	0.594	0.931	0.700
RT	1.000	0.496	0.000	0.106	0.840	0.392	0.303	0.237
RAD	0.000	0.907	0.627	0.641	0.688	0.802	0.968	1.000
BCC	0.000	0.917	0.867	0.794	0.740	0.775	0.853	1.000
RCC	0.000	0.981	0.924	0.850	0.807	0.831	0.884	1.000
BCT	0.000	0.953	0.837	0.747	0.723	0.777	0.832	1.000
RCT	0.000	0.995	0.938	0.859	0.819	0.854	0.893	1.000
TF	0.000	0.958	0.892	0.868	0.882	0.911	0.932	1.000
SOD	0.692	0.768	0.825	0.858	1.000	0.755	0.565	0.000
POD	0.775	0.888	0.899	1.000	0.361	0.171	0.149	0.000
CAT	0.378	0.382	0.698	0.819	1.000	0.479	0.042	0.000
APX	0.551	0.612	0.673	1.000	0.673	0.449	0.122	0.000
GR	0.379	0.494	0.540	1.000	0.460	0.356	0.184	0.000
SP	0.000	0.423	0.563	1.000	0.713	0.646	0.576	0.444
$O_2\cdot^-$	1.000	0.480	0.400	0.360	0.000	0.220	0.280	0.440
MDA	0.000	1.000	0.795	0.360	0.328	0.404	0.443	0.377
H_2O_2	0.415	1.000	0.686	0.415	0.000	0.144	0.051	0.000
Pro	0.000	0.747	0.874	1.000	0.789	0.589	0.411	0.116
·OH	0.058	1.000	0.351	0.221	0.000	0.052	0.221	0.266
GSSG	0.000	0.429	0.571	0.700	1.000	0.743	0.329	0.100
GSH	0.609	0.592	0.793	1.000	0.387	0.194	0.132	0.000
ASA	0.000	0.030	0.424	0.818	1.000	0.545	0.515	0.242
DHA	0.250	0.000	0.917	1.000	0.833	0.583	0.500	0.250
Spd	0.000	0.183	0.254	1.000	0.874	0.774	0.661	0.439
Spm	0.000	0.087	0.143	1.000	0.651	0.253	0.142	0.078
Put	0.000	0.249	0.432	1.000	0.985	0.747	0.537	0.056
MV	0.317	0.512	0.552	0.758	0.668	0.552	0.474	0.354

Rank	8	5	3	1	2	4	6	7

GE: germination energy. GP: germination percentage. GI: germination index. VI: vigor index. GCRI: germination Cd resistance index. VCRI: vigor Cd resistance index. RL: root length. BL: bud length. FW: fresh weight. DW: dry weight. R/S: root-to-shoot ratio. RWC: relative water content. REL: relative electrical conductivity. RSA: root surface area. RV: root volume. RT: number of root tips. RAD: root average diameter. BCC: bud Cd concentration. RCC: root Cd concentration. BCT: bud Cd content. RCT: root Cd content. TF: translocation factor. SOD: superoxide dismutase activity. POD: peroxidase activity. CAT: catalase activity. APX: ascorbate peroxidase activity. GR: glutathione reductase activity. SP: soluble protein. $O_2 \cdot^{-}$: superoxide anion rate. MDA: malondialdehyde. H_2O_2: hydrogen peroxide. Pro: free proline. ·OH: hydroxyl radical. GSSG: oxidized glutathione. GSH: reduced glutathione. AsA: ascorbic acid. DHA: dehydroascorbic acid. Spd: spermidine. Spm: spermine. Put: putrescine.

Table S4 Comprehensive analysis of the effects of different concentrations of exogenous Put on Chang 6475 under Cd stress

**Index**	**CK**	**T0**	**T1**	**T2**	**T3**	**T4**	**T5**	**T6**
GE	0.000	0.775	0.825	1.000	0.825	0.900	0.875	0.900
GP	0.125	0.625	0.625	1.000	0.000	0.375	0.250	0.500
GI	0.000	0.765	0.809	0.986	0.845	1.000	0.895	0.946
VI	1.000	0.000	0.327	0.561	0.572	0.482	0.379	0.346
GCRI	1.000	0.361	0.333	0.917	0.403	0.222	0.000	0.083
VCRI	0.579	0.402	0.533	1.000	0.486	0.410	0.322	0.000
RL	1.000	0.000	0.303	0.443	0.473	0.439	0.395	0.254
BL	1.000	0.000	0.349	0.467	0.591	0.380	0.342	0.268
FW	0.647	0.449	0.520	0.696	1.000	0.591	0.416	0.000
DW	0.828	0.590	0.662	0.819	1.000	0.514	0.320	0.000
R/S	0.745	0.591	0.782	0.625	1.000	0.118	0.021	0.000
RWC	0.604	0.000	0.282	0.827	1.000	0.779	0.813	0.984
REL	0.000	1.000	0.767	0.341	0.132	0.548	0.657	0.964
RSA	0.437	0.000	0.465	0.894	1.000	0.644	0.532	0.241
RV	0.280	0.000	0.413	1.000	0.979	0.879	0.687	0.599
RT	0.415	0.185	0.441	0.547	1.000	0.392	0.282	0.000
RAD	0.000	0.851	0.836	0.832	0.823	0.912	0.949	1.000
BCC	0.000	0.906	0.827	0.773	0.827	0.873	0.924	1.000
RCC	0.000	0.927	0.904	0.846	0.879	0.904	0.946	1.000
BCT	0.000	0.857	0.789	0.749	0.784	0.831	0.908	1.000
RCT	0.000	0.909	0.882	0.849	0.862	0.874	0.922	1.000
TF	0.000	0.943	0.894	0.882	0.910	0.951	0.984	1.000
SOD	0.000	0.557	0.713	0.794	1.000	0.635	0.372	0.202
POD	0.298	0.713	0.881	1.000	0.726	0.458	0.000	0.129
CAT	0.000	0.046	0.184	0.393	1.000	0.301	0.250	0.000
APX	0.241	0.448	0.460	0.621	1.000	0.092	0.046	0.000
GR	0.362	0.448	0.810	1.000	0.509	0.116	0.069	0.000
SP	0.000	0.434	0.636	1.000	0.768	0.631	0.533	0.449
$O_2\cdot^-$	1.000	0.600	0.267	0.000	0.378	0.522	0.056	0.133
MDA	0.367	1.000	0.277	0.108	0.000	0.083	0.566	0.797
H_2O_2	0.000	0.647	0.630	0.341	0.867	1.000	0.519	0.452
Pro	0.000	0.650	0.791	0.907	1.000	0.774	0.612	0.372
·OH	0.000	1.000	0.719	0.456	0.079	0.351	0.474	0.623
GSSG	0.000	0.384	0.444	0.636	1.000	0.697	0.333	0.192
GSH	0.381	0.439	0.529	1.000	0.344	0.344	0.221	0.000
ASA	0.280	0.344	0.598	1.000	0.704	0.598	0.365	0.000
DHA	0.000	0.441	0.559	0.824	1.000	0.912	0.706	0.228
Spd	0.000	0.355	0.603	1.000	0.828	0.499	0.376	0.346
Spm	0.000	0.084	0.185	1.000	0.670	0.353	0.115	0.101
Put	0.000	0.069	0.434	0.827	1.000	0.823	0.692	0.448
MV	0.293	0.501	0.589	0.761	0.719	0.581	0.479	0.414

Rank	8	5	3	1	2	4	6	7

GE: germination energy. GP: germination percentage. GI: germination index. VI: vigor index. GCRI: germination Cd resistance index. VCRI: vigor Cd resistance index. RL: root length. BL: bud length. FW: fresh weight. DW: dry weight. R/S: root-to-shoot ratio. RWC: relative water content. REL: relative electrical conductivity. RSA: root surface area. RV: root volume. RT: number of root tips. RAD: root average diameter. BCC: bud Cd concentration. RCC: root Cd concentration. BCT: bud Cd content. RCT: root Cd content. TF: translocation factor. SOD: superoxide dismutase activity. POD: peroxidase activity. CAT: catalase activity. APX: ascorbate peroxidase activity. GR: glutathione reductase activity. SP: soluble protein. $O_2{\cdot}^-$: superoxide anion rate. MDA: malondialdehyde. H_2O_2: hydrogen peroxide. Pro: free proline. ·OH: hydroxyl radical. GSSG: oxidized glutathione. GSH: reduced glutathione. AsA: ascorbic acid. DHA: dehydroascorbic acid. Spd: spermidine. Spm: spermine. Put: putrescine.
